# Capability instruments in economic evaluations of health-related interventions: a comparative review of the literature

**DOI:** 10.1007/s11136-019-02393-5

**Published:** 2019-12-24

**Authors:** Timea Mariann Helter, Joanna Coast, Agata Łaszewska, Tanja Stamm, Judit Simon

**Affiliations:** 1 Department of Health Economics, Center for Public Health, Medical University of Vienna, Kinderspitalgasse 15, 1090 Vienna, Austria; 2 Health Economics Bristol, Bristol Medical School, University of Bristol, 1-5 Whiteladies Road, Bristol, BS8 1NU UK; 3 Section for Outcomes Research, Center for Medical Statistics, Informatics, and Intelligent Systems, Medical University of Vienna, Spitalgasse 23, 1090 Vienna, Austria; 4 Department of Psychiatry, University of Oxford, Warneford Hospital, Oxford, OX3 7JX UK

**Keywords:** Capability approach, Patient reported outcome measures, Outcome, Validation, Preference weighting, Economic evaluation

## Abstract

**Purpose:**

Given increasing interest in using the capability approach for health economic evaluations and a growing literature, this paper aims to synthesise current information about the characteristics of capability instruments and their application in health economic evaluations.

**Methods:**

A systematic literature review was conducted to assess studies that contained information on the development, psychometric properties and valuation of capability instruments, or their application in economic evaluations.

**Results:**

The review identified 98 studies and 14 instruments for inclusion. There is some evidence on the psychometric properties of most instruments. Most papers found moderate-to-high correlation between health and capability measures, ranging between 0.41 and 0.64. ASCOT, ICECAP-A, -O and -SCM instruments have published valuation sets, most frequently developed using best–worst scaling. Thirteen instruments were originally developed in English and one in Portuguese; however, some translations to other languages are available. Ten economic evaluations using capability instruments were identified. The presentation of results show a lack of consensus regarding the most appropriate way to use capability instruments in economic evaluations with discussion about capability-adjusted life years (CALYs), years of capability equivalence and the trade-off between maximisation of capability versus sufficient capability.

**Conclusion:**

There has been increasing interest in applying the capability-based approach in health economic evaluations, but methodological and conceptual issues remain. There is still a need for direct comparison of the different capability instruments and for clear guidance on when and how they should be used in economic evaluations.

## Background

Economic evaluations assess whether an intervention provides value for money through the comparative analysis of alternative courses of action in terms of both costs and consequences [1]. The assessment of consequences in economic evaluation requires information about their identification (what), measurement (how much) and valuation (how valuable) [2]. Standard methods of health economic evaluations identify outcomes based on a rather narrow definition of health that aims to express outcomes in Quality-Adjusted Life Years (QALYs). However, there are many interventions, particularly in the areas of mental health, end-of-life care, public health and social care, where the impacts of interventions go beyond this narrow view of health. The contemporary literature (e.g. [3–6]) recognises the need to move away from the standard methods for assessing effects of interventions and toward incorporating outcomes beyond the QALY framework, when producing an economic evaluation which feeds into decision making about resource allocation in health-related interventions. The most promising approach to address this issue is the application of Sen’s capability framework, which was introduced by Sen [7] in the early 1980s as an alternative to standard utilitarian welfare economics. The core focus of the capability approach is on what individuals are able to be and do in their lives (i.e. capable of). The application of the capability approach in health economics has gained popularity because it potentially provides a richer evaluative space for the evaluation of interventions [8].

There has been increasing interest in developing instruments for using the capability approach in the measurement and valuation of outcomes for health economic evaluations. Capability instruments have been in the public domain for over a decade and publications have started to shift from methodological issues towards use of the measures within economic evaluations. Some decision-making institutions currently recommend the inclusion of capability measures in economic evaluations in certain contexts. The Zorginstituut in the Netherlands [9] recommends the inclusion of ICEpop CAPability measure for Older people (ICECAP-O) alongside the EuroQol instrument (EQ-5D) for the evaluation of interventions in long-term care, where the relevant outcomes extend beyond health. The most recent methods guideline [10] of the National Institute for Health and Care Excellence (NICE) acknowledges that the intended outcomes of interventions go beyond changes in health status for some decision problems; hence, ‘broader, preference weighted measures of outcomes, based on specific instruments, may be more appropriate…’ and ‘the economic analysis may also consider effects in terms of capability and well-being’ (p. 137). The manual specifically recommends the Adult Social Care Outcomes Toolkit (ASCOT) and ICECAP-O instruments.

However, the choice between instruments and their practical application in particular contexts lack a systematic approach. For instance, the ICECAP-O recommended by NICE is targeted at a subgroup of the population (older adults), whilst the ASCOT was specifically developed for the assessment of social care interventions. A recent review of the literature examined current trends in the application of ICECAP-O [11]. The authors found that the ICECAP-O has mainly been included as a secondary economic measure and the reporting of results is brief with minimal detail and often no discussion or interpretation. An overview of the psychometric properties of all potential capabilities instruments and their usefulness for economic evaluations would contribute to providing a clear guidance. This could later be used as a reference point for future comparative analysis of policies or interventions. Hence, the main aim of this paper is to synthesise the current evidence about the application of capability instruments in health economic evaluations. This translates into the following objectives: (i) to summarise information about the development, psychometric properties and preference valuation of relevant capability instruments; (ii) to compare the identified capability instruments in terms of their psychometric properties and up-to-date application in health economic evaluations; (iii) to identify applied evaluations that have used the capability-based approach in health economic evaluations and (iv) to pinpoint the challenges and considerations in the application of the capability approach in economic evaluations of health-related interventions.

## Methods

### Identification of relevant studies

The identification of papers was based on two main approaches: a traditional systematic literature search and a comprehensive pearl growing method [12]. The grey literature search in Google Advance either generated an unmanageable number of hits due to the term “capability” being used across a number of disciplines with varying meanings, as well as having generic lay use and interpretation of the term; or there was no addition to the search of other databases when more precise terms were used. As the development and validation of the capability approach in health economics currently appears to be concentrated among a limited group of researchers, as an additional step, websites dedicated to the instruments identified through the systematic search were specifically targeted and reviewed for relevant information.

#### Systematic literature search

Firstly, we conducted a systematic literature search. Search terms combined expressions for economic evaluation and frequently used terms for the capability approach, including synonyms and names of instruments most well-known in the area of health economics. Search terms are presented in Appendix 1. The selection of databases was based on similar reviews of health measures (PROMs) [6, 13] in the area and included Embase, Medline, Web of Science, Psychinfo and Scopus. The literature search was conducted on 1 February 2019 and the review was limited to the last 20 years when the first publications in this topic area appeared [14]. Relevant systematic literature reviews were searched for further references and their findings were kept for comparison and discussion.

#### Comprehensive pearl growing method

The term ‘capability’ produces very broad ranging results when used as a search term due to its wide range of meanings, including lay meanings. The so-called comprehensive pearl growing method [12] is a technique used to ensure all relevant articles are included, particularly in case of issues with vocabulary in a search strategy. This method is particularly useful in interdisciplinary research and where recent developments are expected in the literature. The process of pearl growing commences with the identification of ‘key pearls’ (i.e. key studies), that can be identified from within the literature as being compatible with the aim of the review [12]. Once the key pearls have been identified, these are used to generate the ‘first wave of pearls’, that is, papers that have cited the key pearls within their reference list. It has been used successfully in a different type of review in the context of capabilities [13]. This second approach was implemented to validate the strategy applied during the systematic search and to identify potential further papers.

Two waves of the pearl growing method were conducted: one focusing on the development of instruments and a second wave related to the application of the instruments. A third wave was deemed unnecessary because the identified last generation of seminal papers were published only recently and have not been cited yet. The results are presented in Table 1. The first wave used for citation searching were the developmental studies of the four most commonly used and reported capability instruments: ASCOT, ICECAP-O, its version for adults (ICECAP-A) and the Oxford CAPabilities questionnaire-Mental Health (OxCAP-MH). The second wave relied on the three main papers from the last 5 years (but already with some relevant citations) that aimed to identify recent developments and up-to-date knowledge in the application of the capability approach in health economic evaluations. The number of citations was retrieved from Scopus on 14 March 2019.

**Table 1:** Key pearls for the two waves of the comprehensive pearl growing method

Wave	Study	Number of citations	Short description
Wave 1	[52]	92	Development of the ASCOT
	[53]	146	Development of the ICECAP-A
	[54]	158	Development of the ICECAP-O
	[39]	66	Development of the OxCAP-MH
Wave 2	[48]	27	Description of new methods to conduct economic evaluations using the capability approach
	[55]	13	Presents the opportunities and challenges of the capability approach in health economics
	[49]	4	Critical review of relevant questionnaires to measure and value capability

### Study selection

Titles and abstracts were sifted by two researchers (TL and AL) and studies were included for further assessment if they met the following inclusion criteria: (1) Full paper available in *English or German* languages. (2) Scope of study is the a*rea of health or health*-*related interventions*, including any interventions specifically targeting the promotion of health and prevention and treatment of ill-health irrespective of the sector where these were implemented. Hence, our study also included potentially relevant studies from the social care and public health sectors. (3) Focus of research is the evaluation or assessment of the outcomes of interventions using the *capability approach.* (4) Paper includes information on the use (or recommended use) of the capability approach in *economic evaluations*. (5) Paper is an *applied evaluation* OR focuses on the *development, psychometric validation* (or comparison to other tools)* or preference valuation* of instruments.

The full paper was retrieved if a study met the inclusion criteria based on its title and abstract. Consequently, full papers were assessed by two researchers (TH and AL) for inclusion based on their contribution to at least one of the aims of this literature review and subsequently allocated to the categories of either (i) applied evaluations (using a capability instrument in a completed economic evaluation) or (ii, iii, iv, v) methods papers. Methods papers were further categorised based on their relevance to the identification, measurement and valuation of outcomes, as well as the practical application of tools and theoretical contributions. Papers were grouped into categories of (ii) instrument development, (iii) psychometric validation or quantitative comparison of instruments, (iv) preference valuation of instruments and (v) methods for incorporation of the capability approach in economic evaluations. The latter one includes potential fields of application, approaches to use the results, incorporation of the results into a potential framework, for instance, Capability-Adjusted Life Years (CALYs), years of full capability or years of sufficient capability equivalence, etc. Some of the studies with significant theoretical contributions to the application of the capability approach in health economic evaluations which did not fit the above criteria were noted for discussion.

No specific quality assessment was applied, all studies which provided information on either the psychometric properties or use of capabilities instruments in economic evaluations were included. The instruments were assessed based on their psychometric properties according to the COSMIN checklist [15], feasibility [16], potential for transferability and evidence regarding valuation.

### Data extraction and analysis

Separate data extraction forms were created for empirical and psychometric evaluation (and other methods) studies. The search for information on valuation included any kind of preference-based valuation of instruments (or their dimensions/domains) and the existence of value sets. Further information on data extraction is presented in Appendix 2.

Trends in the literature were analysed based on the number of different types of studies published each year. The information elicited from the studies was structured according to the capability instrument in question. Information about economic evaluations, and the psychometric properties and correlation coefficients from studies comparing instruments are presented in review tables. Due to the variability of methods used in the validation and comparison studies, only narrative synthesis, including tabulation and frequency analyses, was conducted as no statistical pooling was possible. The information gathered was synthesised in a qualitative rather than quantitative manner by TH.

## Results

### Search results

**Fig. 1:**
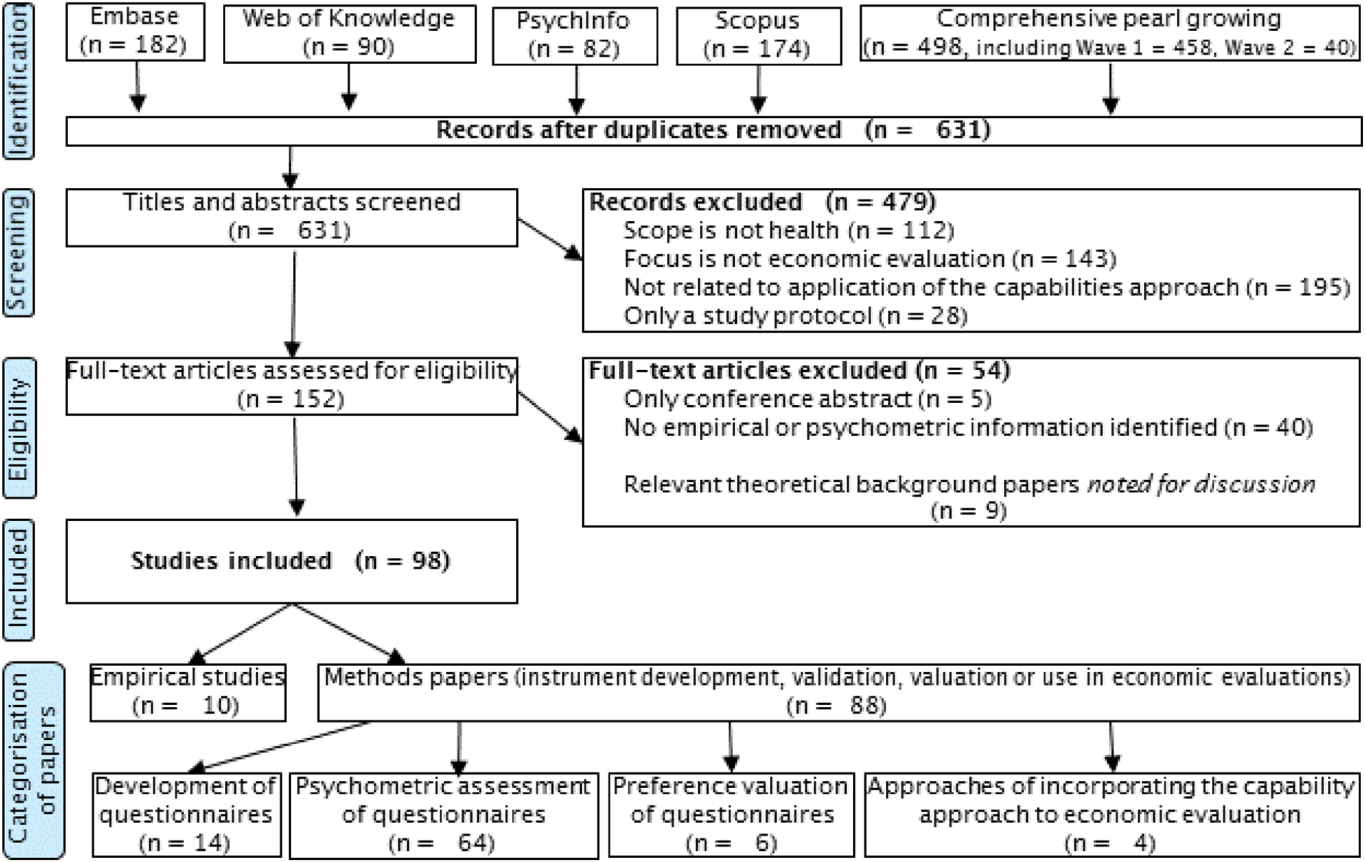
PRISMA chart

The literature search identified 98 studies for inclusion (Appendix 4 provides a complete list). The pearl growing method identified 29 citations beyond those captured by the systematic search strategy. However, none of the additional references met the inclusion criteria, and the papers included in this review were actually all picked up by the systematic search. An overview of the literature search based on the PRISMA statement is presented in Fig. 1.

**Fig. 2:**
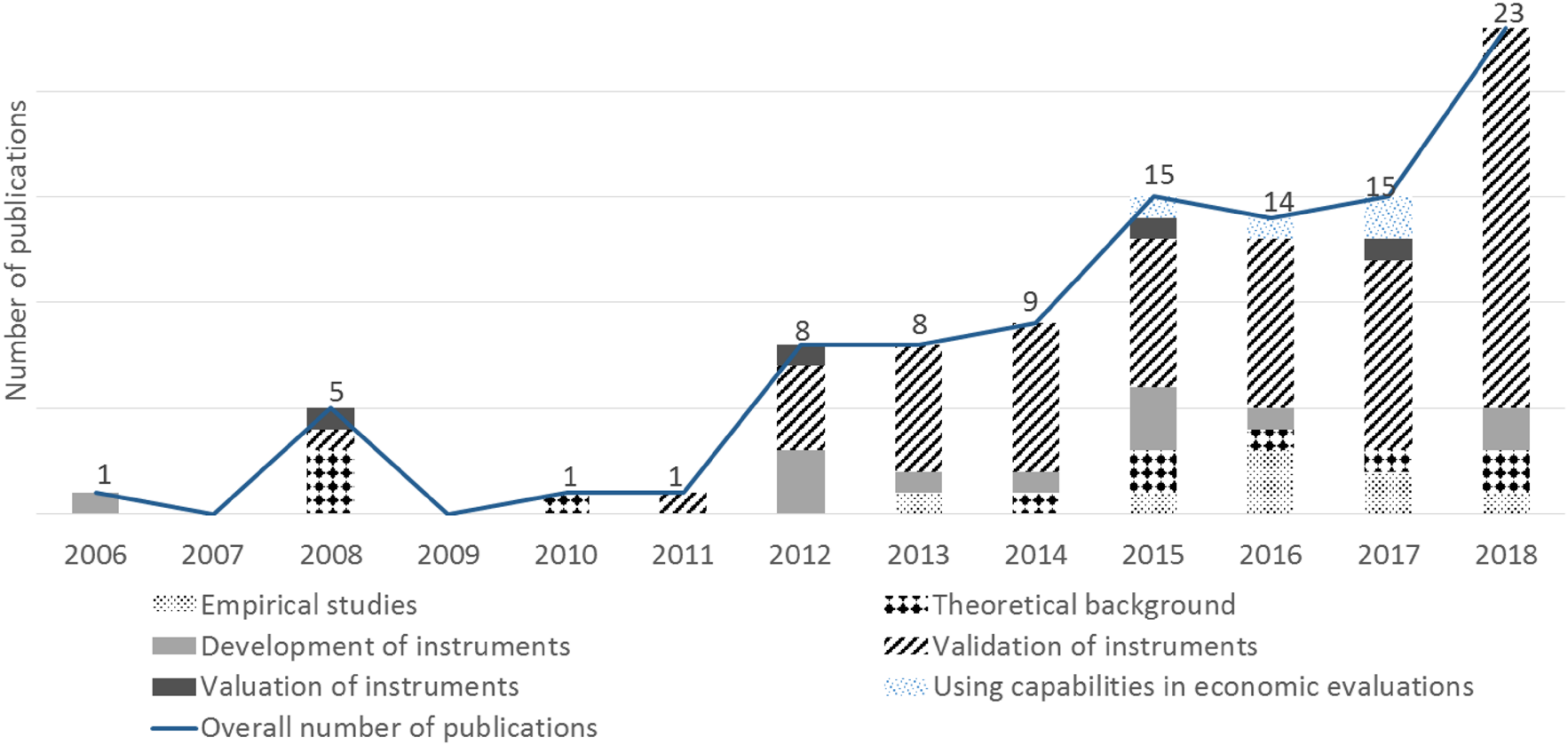
Annual changes in the number and type of publications related to using the capability approach in the economic evaluation of health-related interventions. Year 2019 not included in this figure because data were not available for the full year. Instruments to assess capability

The increasing number of relevant publications in recent years is a clear trend (shown in Fig. 2). A further trend also appears to be a shift from developmental studies towards the validation of capability instruments and their use in empirical studies.

### Instruments to assess capability

#### Development of instruments

The literature review identified 14 capability instruments. Table 2 shows the heterogeneity of the capability instruments in terms of development methods, disease areas, types of interventions, population groups and the questionnaire structure.

**Table 2:** Overview of the main characteristics and development methods of instruments that measure and value capability for economic evaluations in health

Instrument	Instrument full name	Field	Population	Number of		Development method	Informants	Number of informants	References
				Dimensions	Levels				
ACQ‐CMH‐104	Achieved Capabilities Questionnaire for Community Mental Health	Mental health	Patients	104	Unknown	Focus groups	Participants of Portuguese community mental health services	50	[56]
ASCOT	Adult Social Care Outcomes Toolkit	Social care	Patients	8	4	Delphi exercise, Literature review and expert opinion, *Further improvement of the Older People’s Utility Scale (OPUS)*	Experts and service users	330	[52]
ASCOT Easy Read version	Easy Read Version of the Adult Social Care Outcomes Toolkit	Social care	People w. intellectual disabilities	8	4	Focus groups and in-depth interviews	Intellectual disability or autism	54	[57]
ASCOT – proxy version	Proxy-report version of the Adult Social Care Outcomes Toolkit	Social care	Patients	8	4	In-depth qualitative interviews	Adult care workers or unpaid family carers of patients with cognitive and/or communication impairments	25	[58]
ASCOT-Carer	Carer Version of Adult Social Care Outcomes Toolkit	Social care	Carers	7	4	Literature review and feedback from service users, carers, practitioners and policy-makers	Service users, carers, practitioners and policy-makers	31	[59]
CAF	Currently Achieved Functioning	General	Older people	5	5	In-depth qualitative interviews	Older people living in 3 Dutch urban areas	99	[60]
ICECAP-A	ICEpop CAPability measure for Adults	General	General public	5	4	In-depth, informant-led, interviews	General public (purposively selected through a random electoral sample)	36	[53]
ICECAP-CPM	ICEpop CAPability Close Person Measure	End of life	Close persons	6	5	In-depth qualitative interviews	Bereaved within the last 2 years or with a close person currently receiving end-of-life care	27	[61]
ICECAP-O	ICEpop CAPability measure for Older people	General	Older people	5	4	In-depth qualitative interviews	Purposively selected informants aged 65 and over in private households	40	[54]
ICECAP-SCM	ICEpop CAPability Supportive Care Measure	End of life	Patients	7	4	In-depth qualitative interviews	Older people from different groups across the dying trajectory	23	[62]
OCAP-18	OCAP-18	Public health	General public	18	Unknown	Theoretical framework, Focus groups and in-depth interviews	Purposively sampled from various community groups in Glasgow, United Kingdom	40	[63]
OxCAP-MH	Oxford Capabilities Questionnaire for Mental Health	Mental health	Patients	16	5	Theoretical framework, Focus group discussions	Psychiatrists, Psychologists, Social scientists, Health economists	336	[39]
(Low-income questionnaire)	(Low-income questionnaire)	Low-income settings	General public	6	Unknown	Focus groups	Women in rural Malawi	129	[64]
(Chronic pain questionnaire)	(Chronic pain questionnaire)	Chronic pain	Patients	8	Unknown	Focus groups and in-depth interviews	Respondents were recruited through a Pain Management Clinic in the East of England	16	[65]

#### Availability of evidence on the characteristics of capability instruments

As Table 3 demonstrates, there is at least some evidence about the psychometric properties of most instruments.

**Table 3:** Availability of evidence on the characteristics of capability instruments for health economic evaluations

Instrument	Reliability	Validity	Responsiveness	Interpretability/Feasibility	Valuation
ACQ‐CMH‐104	[66]	[66]	Unknown	Unknown	Unknown
ASCOT	[67]	[21, 68, 69, 70, 71, 72, 73, 74]	[71]	[75]	[52]
ASCOT easy read	Unknown	Unknown	Unknown	[57]	Unknown
ASCOT-proxy	Unknown	Unknown	Unknown	[58]	Unknown
ASCOT-carer	[76]	[76]	Unknown	Unknown	Unknown
CAF	Unknown	Unknown	Unknown	[60]	Unknown
ICECAP-A	[77]	[20, 23, 24, 27, 33, 34, 38, 78, 79, 80]	[23, 32, 33, 34, 37, 81]	[82, 83, 84]	[85]
ICECAP-CPM	Unknown	Unknown	Unknown	Unknown	Unknown
ICECAP-O	[30, 86, 87]	[18, 21, 22, 25, 26, 40, 74, 87, 88, 89, 90, 91, 92, 93, 94, 95, 96]	[26, 31, 35, 36, 95, 97]	[25, 26, 30, 40, 89, 91, 98, 99]	[88]
ICECAP-SCM	Unknown	Unknown	Unknown	[29, 83]	[100, 101]
low-income Q	Unknown	[102]	Unknown	Unknown	Unknown
pain Q	Unknown	Unknown	Unknown	Unknown	Unknown
OCAP-18	Unknown	Unknown	Unknown	Unknown	Unknown
OxCAP-MH	[17, 19, 103]	[17, 19, 103]	[17]	[39]	Unknown

The most recently developed instruments, unsurprisingly, have less information available about their reliability, validity and responsiveness; an exception is OCAP-18 which was among the first capability instruments to be developed, but for which there is no further psychometric evidence available. The main difference across different groups of capability instruments is whether valuations that reflect the preferences of patients or the general public are available. The ASCOT and most ICECAP instruments have reported valuation studies and are therefore considered to possess evidence regarding their ability to reflect values of informants, whilst this is currently missing, for instance, for OxCAP-MH.

#### Different language versions of instruments

Apart from ACQ‐CMH‐104, all instruments were originally developed in English. The ASCOT, ICECAP-A, ICECAP-O and OxCAP-MH instruments have been translated to further languages, and these new versions have been validated (Table 4).

**Table 4:** Availability of different language versions of capability instruments

Instrument	Availability of language versions beside English^a^
ACQ‐CMH‐104	Only available in Portuguese language
ASCOT	Japanese [105]; Dutch [106]
ASCOT easy read	None identified
ASCOT-proxy	None identified
ASCOT-carer	None identified
CAF	None identified
ICECAP-A	Chinese [107], Danish (unpublished), Dutch (unpublished), German [107], Italian (unpublished), Persian (unpublished), Welsh (unpublished)
ICECAP-CPM	none identified
ICECAP-O	Chinese (unpublished), Dutch [92], French (unpublished), German [18], Spanish [87], Swedish [86], Welsh (unpublished); Italian, Norwegian and Portuguese [109]
ICECAP-SCM	None identified
low-income Q	None identified
pain Q	None identified
OCAP-18	None identified
OxCAP-MH	German [103]

^a^Information on unpublished translations of instruments stem from the dedicated websites of the instruments

#### Validation of capability instruments

##### Reliability

The test–retest reliability of most instruments have been successfully assessed in some groups of population, e.g. ACQ‐CMH‐104 [56]; ASCOT [72]; ICECAP-A [77]; ICECAP-O [86]; OxCAP-MH [19].

##### Validity

There were 25 studies among the included papers that used Pearson’s or Spearman rank correlation coefficients to quantitatively assess the validity of all language versions of the capability instruments and/or compare it to other instruments. Quantitative evidence was provided on the validity of six capability instruments, including ACQ‐CMH‐104, ASCOT, ICECAP-A, ICECAP-O, OxCAP-MH and Women’s Capabilities Index. Table 5 (and Appendix 5) summarise the correlations.

**Table 5:** Construct validity of capability instruments for health economic evaluations

Capabilities instrument	Compared with… (full names in Appendix 5)	Value of correlation*	Population (country in Appendix 5)	Number of informants	References
ACQ‐CMH‐104	RAS	0.46*	Psychiatric patients	92	[66]
	WHOQOL‐Bref	0.60*	Psychiatric patients	129	[66]
ASCOT	Barthel Index	0.45	Older social care users	205	[21]
	Cantril’s Ladder	*0.*66	Older social care users	205	[21]
	CASP-12	*0.58*	Older home care residents	301	[52]
	EQ-5D-3L	*0.41*	Older home care residents	301	[52]
	EQ-5D-3L	*0.40*	Older home care residents	301	[70]
	EQ-5D-3L	*0.*47	Older home care residents	224	[68]
	EQ-5D-3L	0.41*	Frail older adults living at home	190	[74]
	EQ-5D-3L	0.37	Older social care users	748	[72]
	EQ-5D-5L	0.63	Older social care users	205	[21]
	EQ-5D-5L	0.24	Older adults in a day rehabilitation facility	22	[71]
	EQ-5D-VAS	0.64	Older social care users	205	[21]
	GDS-15	− 0.69	Older social care users	205	[21]
	GHQ-12	− *0.58*	Older home care residents	301	[52]
	ICECAP-A	0.62	Older social care users	748	[72]
	ICECAP-O	0.81	Older social care users	205	[21]
	ICECAP-O	0.41*	Frail older adults living at home	190	[74]
	ICECAP-O	0.67	Older social care users	748	[72]
	OPQOL-13	0.76	Older social care users	205	[21]
	OPQOL-brief	0.38	Older adults in a day rehabilitation facility	22	[71]
	OPQoL-Brief	0.58	Older social care users	87	[69]
	SWLS	0.74	Older social care users	205	[21]
ASCOT-Carer	CES	0.58	Social care recipients	376	[76]
	CSI	− 0.59	Social care recipients	384	[76]
	EQ-5D-3L	0.34	Social care recipients	382	[76]
	QoL	0.62	Social care recipients	384	[76]
ICECAP-A	15D	0.50*	Healthy general public and patients from 8 disease areas	6756	[24]
	AQoL-8D	0.31*	Healthy general public and patients from 8 disease areas	6756	[24]
	AQoL-8D	0.80	Healthy general public and patients with 7 chronic conditions	8022	[20]
	EQ-5D-3L	*0.53*	Women with lower urinary tract infection	478	[23]
	EQ-5D-3L	0.49	Knee pain patients in primary care	500	[27]
	EQ-5D-5L	0.62*	Healthy general public and patients with 7 chronic conditions	1212	[108]
	EQ-5D-5L	0.49*	Healthy general public and patients from 8 disease areas	6756	[24]
	EQ-5D-5L	0.60	Healthy general public and patients with 7 chronic conditions	8022	[20]
	HUI-3	0.32*	Healthy general public and patients from 8 disease areas	6756	[24]
	LDQ	− *0.48*	Opiate substitution recipients	83	[34]
	SF-6D	0.64*	Healthy general public and patients with 7 chronic conditions	1212	[108]
	SF-6D	0.47*	Healthy general public and patients from 8 disease areas	6756	[24]
	SSQ	*0.43*	Opiate substitution recipients	83	[34]
	SWLS	0.66*	Healthy general public and patients with 7 chronic conditions	1212	[108]
ICECAP-O	ADRQL	0.53*	Nursing home residents with dementia	95	[18]
	Barthel Index	0.49	Older social care users	209	[21]
	Barthel Index	0.72*	Nursing home residents with dementia	95	[18]
	Cantril’s Ladder	0.74	Older social care users	213	[21]
	CTM-3	0.23	Patients from outpatient day rehabilitation unit	82	[22]
	EQ-5D-3L	*0.34*	Older people with hip fracture	113	[95]
	EQ-5D-3L	0.69*	Nursing home residents with dementia	95	[18]
	EQ-5D-3L	0.53	Older people after hip fracture surgery	87	[93]
	EQ-5D-3L	0.44	Patients from outpatient day rehabilitation unit	80	[22]
	EQ-5D-3L	0.47	Patients visiting the clinic	215	[25]
	EQ-5D-3L	0.63	Frail older adults living at home	190	[74]
	EQ-5D-5L	0.68	Older social care users	207	[21]
	EQ-5D-5L	0.63	General population aged 70 or older	516	[90]
	EQ-5D-VAS	0.66	Older social care users	208	[21]
	GDS-15	− 0.73	Older social care users	210	[21]
	OHS	*0.38*	Older people with hip fracture	113	[95]
	OPQOL-13	0.80	Older social care users	211	[21]
	SWLS	0.82	Older social care users	212	[21]
ICECAP-O family version	EQ-5D family version	0.57*	Nursing professionals of psycho-geriatric elderly	96	[92]
	EQ-VAS family version	0.43*	Family members of psycho-geriatric elderly	68	[92]
ICECAP-O nursing version	EQ-5D nursing version	0.48*	Nursing professionals of psycho-geriatric elderly	96	[92]
	EQ-VAS nursing version	0.55*	Family members of psycho-geriatric elderly	68	[92]
OxCAP-MH	BPRS	− *0.41*	Patients with psychosis	172	[19]
	BSI-18	− 0.67*	Patients in socio-psychiatric services	162	[17]
	EQ-5D VAS	0.58*	Patients in socio-psychiatric services	161	[17]
	EQ-5D-3L	*0.45*	Patients with psychosis	172	[19]
	EQ-5D-5L	0.66*	Patients in socio-psychiatric services	160	[17]
	EQ-5D-VAS	*0.52*	Patients with psychosis	172	[19]
	GAF	*0.24*	Patients with psychosis	172	[19]
	GAF	0.35*	Patients in socio-psychiatric services	168	[17]
	Mini-ICF-APP	− *0.*47*	Patients in socio-psychiatric services	167	[17]
	SIX	*0.12*	Patients with psychosis	172	[19]
	WHOQOL-Bref Environment	0.69*	Patients in socio-psychiatric services	166	[17]
	WHOQOL-BREF Physical health	0.69*	Patients in socio-psychiatric services	163	[17]
	WHOQOL-Bref Psychological	0.75*	Patients in socio-psychiatric services	164	[17]
	WHOQOL-Bref Social relationships	*0.*50	Patients in socio-psychiatric services	165	[17]
Women’s Capabilities Index	WHOQOL-Bref	0.62*	Women from Malawi	20	[64]

Values in *italic* are Pearson’s coefficients, values in standard writing are Spearman rank correlations. A * behind the value means that the study used a non-English version of the capability instrument

There is variation between studies in the correlation measures used, the instruments compared, the characteristics of the population, number of informants, testing of hypotheses generated regarding likely associations between the data and testing across known groups for discriminant and convergent validity. Hence, it is difficult to provide general statements about the comparison of capability instruments with other PROMs, or to conduct statistical pooling of the results. High correlation estimates (above 0.8) were found between capability instruments: ASCOT/ICECAP-O [49] and ICECAP-A/AQoL-8D [20].

The examined studies provided very diverse estimates for the correlations between Health-related Quality of Life (HRQoL) and the different capability instruments. Most studies compared the ASCOT, ICECAP-A and ICECAP-O instruments with either disease-specific or generic HRQoL instruments. A wide range of disease-specific instruments were applied across studies, mainly being used when informants consisted of patients and social care recipients. EQ-5D-3L/-5L was used in 92% (*n* = 23) of the included validation and comparison studies as a HRQoL measure. In most cases, the 5L version of the EQ-5D instruments provided higher correlation coefficients compared to the 3L version. The higher correlation with capability instruments could be explained by lower ceiling effects and higher sensitivity to minor changes in the 5L version compared to the 3L version.

There seem to be a consensus in the literature that the capability approach provides complementary information to HRQoL measures. However, capability instruments could also be perceived as enhanced rather than complementary to the narrow interpretation of well-being/quality of life when focusing only on HRQoL. Most studies [25–27] found that the ICECAP and EQ-5D instruments provide complementary information, and a mapping is not recommended between them. Engel et al. [24] found that the ICECAP-A provides evidence above that gathered from most commonly used preference-based HRQoL instruments. Similar findings were reported for other capability instruments. Forder and Caiels [68] found that ASCOT has greater validity in measuring the effects of social care services than EQ-5D. Van Leeuwen et al. [28] investigated the validity of ICECAP-O and ASCOT among Dutch older adults. Although it could be attributable to cultural transferability issues, they found that respondents did not feel that these instruments give a comprehensive picture of their HRQoL because they did not find all domains of the instruments relevant, whilst other important domains were not covered, particularly concerns or delight about the well-being of family members. HRQoL instruments capture an important part of broader well-being, and some studies [22, 23] established strong and positive association between capability and HRQoL instruments, which questions whether they focus on complementary constructs. Evidence suggests that some capability instruments could rather be interpreted as an enhancement of the HRQoL concept, for instance, an exploratory factor analysis [17] found that all EQ-5D-5L items and seven OxCAP-MH items loaded on one factor and nine remaining OxCAP-MH items loaded on a separate factor.

It is questionable whether the issues discussed above relate to all HRQoL measures or only the EQ-5D Utility instrument. Lower correlation between the OxCAP-MH and EQ-5D Utility scores was observed in the Vergunst et al. [19] study than between OxCAP-MH and EQ-5D-VAS. This could be explained by the fact that the latter reflects the patient’s overall judgement about their health status rather than focusing only five dimensions of their health, which is arguably more in line with the underlying broader well-being concept and the used non-preference-based index score of the OxCAP-MH instrument.

##### Interpretability

In terms of ease of understanding, Bailey et al. [29] investigated the appropriateness of ICECAP-SCM to measure QoL and found that the capability instrument appeared more meaningful, easier to complete and had fewer errors among patients and close persons, compared to EQ-5D-5L. However, these results did not apply to healthcare professionals who preferred the EQ-5D-5L over ICECAP-SCM when measuring clinician-rated health states because it focused on observable attributes. Similar studies have also demonstrated the feasibility of use of other ICECAP measures [81, 90]. Malley et al. [70] and Towers et al. [67] demonstrated the feasibility of using ASCOT among older people and care home residents; however, the study also highlighted the need for proxy respondents in some situations. This later led to the development of a proxy version of the ASCOT, which demonstrated good feasibility [58]. Davis et al. [30] reported that the level of agreement between patient and proxy for the EQ-5D-3L was significantly better than the level of agreement observed for the ICECAP-O in case of patients with vascular cognitive impairment. The authors conclude that due to its complexity, the ICECAP-O may have limited clinical, research and policy-related utility among individuals with mild cognitive impairment. However, these results need to be interpreted carefully due to the differing number of levels and the greater ability of proxies to observe the dimensions in EQ-5D. Although it could be explained by translational issues, van Leeuwen [28] who also reported difficulties with understanding the ASCOT and ICECAP-O in a study assessing a small number (*n* = 10) of Dutch, community-dwelling frail older adults. Simon et al. [39] explored the feasibility of OxCAP-MH among severely ill mental health service users. Patients provided positive feedback and felt that the questions allowed them to express their views and experience on topics they considered important but which were often left out of clinical or research interviews [39].

##### Responsiveness

The sensitivity of the capability instruments to measure changes is generally reported to be higher than in case of HRQoL measures [6, 17, 31–34]. However, some authors found capability instruments to be less responsive than HRQoL measures. Davis et al. [35] and Couzner et al. [36] reported that the difference in values between the patient and general population groups was found to be far more pronounced for the EQ-5D-3L than for the ICECAP-O. There is a consensus in the literature that changes related to the broader meaning of health are better captured by the capability instruments than by EQ-5D [37–39]. Coast et al. [40] found strong evidence of association of general health with all capability attributes except for the attachment domain of ICECAP-A. Laszewska et al. [17] found that the OxCAP-MH may be seen as enhanced rather than complementary in its concept, when compared to EQ-5D-5L.

#### Valuation of instruments

From the reviewed 14 capability instruments, only four have a published valuation set. These used the best–worst scaling method, most often relying on the MaxDiff model. Informants mainly came from the general public. There is no published evidence available for the valuation of the remaining ten capability questionnaires (Table 6).

**Table 6:** Valuation of capability instruments for health economic evaluations

Instrument	Methods of valuation	Number of choices per BWS task	Number of BWS tasks per respondents	Population	Number of informants	References
ASCOT	BWS, TTO	4	8	General public	958 (BWS) + 126 (TTO)	[52]
ICECAP-A	BWS	5	16	General public	413	[85]
ICECAP-O	Variants of DCEs and BWS tasks (online)	5	16	General public aged 65 or over	255	[88]
ICECAP-SCM	BWS	7	16	General public	6020	[101, 110]

### Applied economic evaluations and potential methods to incorporate the capability approach

Ten applied evaluations were identified in this review that have used a capability-based instrument as secondary outcome measure in health economic evaluations. No economic evaluation was found where a capability instrument was used as a primary measure of health outcomes. The information extracted from the applied evaluations is presented in Table 7 and in Appendix 6.

**Table 7:** Applied evaluations using the capability approach in their economic evaluations

Capability measure	Disease	Time points	Other HE measures	Changes in QALYs vs. capability values	Presentation of results	Reference
ICECAP-A	Visual impairment	Baseline; 2–4 months	EQ-5D-5L	Nearly identical^a^	Cost per Year of Full Capability (YFC)	[111]
	Diabetic plantar ulceration	Baseline; 6 months	EQ-5D-5L	QALYs negative; Capability positive	Cost and outcome data presented separately	[43]
	Drug addiction	Baseline; 12 months	EQ-5D-5L	Full capability higher than Sufficient capability, and both higher than QALYs	Years of full capability (YFC), years of sufficient capability equivalent (YSC)	[112]
	Schizophrenia	Baseline; 12–36–48 weeks	EQ-5D-3L	Nearly identical^a^	Cost and outcome data presented separately	[44]
ICECAP-O	Health decline in the older people	Baseline; 3 months	EQ-5D-3L	QALYs positive; Capability negative	Incremental net monetary benefit (INMB) regressions based on capability QALYs	[31]
	Heart failure, chronic obstructive pulmonary disease, or diabetes	Baseline; 12 months	EQ-5D-3L	Nearly identical^a^	Willingness to pay for 100% improvement in capability	[113]
	Visual impairment	3 months; post-intervention; pre-study	EQ-5D-5L	Capability higher than QALYs	Costs per years of well-being	[46]
	Hip fracture	Baseline; 3 months	EQ-5D-3L	Capability lower than QALYs	Cost and outcome data presented separately	[42]
OxCAP-MH	Psychosis	Baseline; 6–12 months	EQ-5D-3L	Nearly identical^a^	Cost and outcome data presented separately	[45]
ICECAP-A and OxCAP-MH	Schizophrenia or schizoaffective disorder and depression	Baseline, 3–6–9 months	EQ-5D-5L	QALYs positive; Capability: no significant change	Cost and outcome data presented separately	[114]

^a^Nearly identical means that the difference between baseline and follow-up are within a 10% range when comparing the QALYs and capability estimates

The number of economic evaluations reporting the use of a capability instrument has increased in recent years and further increases can be expected given that this search identified a number of recent study protocols (e.g. [41, 42, 114]). Four further studies were identified that specifically addressed the issues and discussed considerations when incorporating the capability approach into health-related economic evaluations.

A recent review [13] focused on using the capability approach in health research, not limited to economic evaluations. It identified four distinct common *areas of application* including: (1) physical activity and diet; (2) patient empowerment; (3) multidimensional poverty and (4) assessments of health and social care interventions. The authors also noted that there is a noticeable non-reliance on health status as a sole indicator of capability in health, and differences were found across studies in approaches to applying mixed methods, selecting capability dimensions and weighting capabilities. The current review identified applied economic evaluations from areas with widely accepted issues related to outcomes beyond the QALYs framework, e.g. mental health, visual impairment, chronic diseases and health decline in older people.

The presentation of results in the included economic evaluations demonstrate that there is a lack of consensus regarding the most appropriate way to use capability instruments in economic evaluations. Some authors present cost and outcome data separately and conduct a cost-consequence analysis [42–45], whilst others reported the results following the idea behind the incremental cost-effectiveness ratio (ICER) [31, 46]. This lack of consensus about the use of capability instruments in decision making relates to the different approaches taken by different research groups to valuation, which means that in practice these measures are not comparable along the lines of a QALY. The idea of CALYs has been proposed by Mansdotter et al. [47] who highlights the following issues. First, it is questionable which capabilities are able to explain differences in well-being and are sensitive to public policies *in high-income countries*. Second, questions of the relevant instruments should capture *voluntary and involuntary positions* because an applied conceptualization of the capability approach includes opportunity as well as achievement. Third, methods for *weighting* capability and *threshold* values should be established, similar to QALYs. Finally, a trade-off should be made between the *maximisation of capability and equity*.

Mitchell et al. [48] proposed the concept of years of sufficient capability which is more closely aligned to the theory underpinning the capability approach because it has a greater focus on those in capability poverty. The process of defining a threshold for sufficient capability should be based on generating a sufficient capability score and using these scores to produce a capability outcome over time [48]. The use of ICECAP-A in the economic evaluations included in this literature review seem to focus on the choice between the options of years of full capability vs. years of sufficient capability equivalent [48].

The current state of the art identified in the reported economic evaluations applying the capability approach to their assessment are in line with the previously identified main challenges [50], including the need to research what the value of a capability improvement is, how to use the instruments globally, and compare the sensitivity of each measure to different patient groups and conditions. Only one study [49] was identified that posed a critique to using the capability approach in health economic evaluations. The authors claim that the method used in the questionnaires to measure capability will result in a capability set that is an inaccurate description of the individual’s true capability set. The measured capability set will either *represent only one combination* and ignore the value of choice in the capability set, or represent *one combination that is not actually achievable* by the individual. In addition, existing methods of valuing capability may be inadequate because they do not consider that capability is a set. (Although the Oxford instruments were developed based on Nussbaum’s 10 basic human capabilities.) Hence, it may be practically more feasible to measure and value capability approximately rather than directly. Nevertheless, the argument is based on the questionable assumption that all capabilities have to be traded against other capabilities.

## Discussion

This systematic literature review about capability instruments in economic evaluations of health-related interventions included 98 articles and identified 14 capability-based instruments. It provides a unique, comprehensive synthesis of the relevant evidence by focusing on the full spectrum of potentially available capability measures and summarising the practical and theoretical aspects of use of these instruments in economic evaluations. Most identified information related to the ASCOT, ICECAP-A, ICECAP-O and OxCAP-MH instruments.

The development of capability instruments relies on methods similar to those applied in the case of HRQoL measures. Capability instruments were often compared to EQ-5D, but less often to each other. Possible reasons for this are that some instruments are population or disease-specific, and that the inclusion of two instruments measuring the same concept in an applied evaluation study is assumed to unnecessarily increase participants’ completion burden. In general, the information identified in the literature regarding the comparison of capability measures with other instruments could not be used for a pooled analysis. This is mainly due to the vast variation in the correlation measures used, the instruments compared, the characteristics of the populations and the number of informants. Despite the diverse quantitative estimates for the correlations with EQ-5D, the different capability instruments and the limited available data, this review confirms that capability measures capture a wider range of outcomes than the EQ-5D and may be more responsive when an intervention is likely to have broad impacts on HRQoL. Following the guidelines [51] to evaluate the strength of correlations, this generally observed moderate-to-high correlation suggests that EQ-5D and capability instruments measure somewhat similar, yet complementary concepts. However, there are competing statements in the literature regarding the association between capability and HRQoL instruments. Most authors argue that these measures complement each other; however, some studies suggest that capability instruments could be perceived as enhancements of the HRQoL concept. It is possible that this relationship depends on the choice of both capability and health instruments used in these comparisons. For instance, the OxCAP-MH has a relatively high number of items, which potentially capture a broader range of capability concepts than measures such as the ICECAP measures. Similarly, the EQ-5D measure of health has a narrower focus than other health measures such as measures based on SF-36 or the AQoL. The higher correlations between capability instruments and the EQ-5D-VAS scores than those observed between capability instruments and the EQ-5D utility scores suggest that respondents’ overall judgement of their health status on a VAS seems to reflect better broader quality-of-life concepts present in the capability approach than specific scores for a certain limited number of HRQoL dimensions. Moreover, the differences in correlations found between measures may be due to differences in the populations studied. Hence, further research could explore which population subgroups and disease areas could benefit from the inclusion of certain capability instruments in economic evaluations.

Three of the identified 14 capability instruments were used in applied economic evaluation of interventions in the health and social care field; however, only as secondary outcome measures. Eight of the identified ten applied economic evaluations were conducted in the United Kingdom. This may be the result of the fact that the measures were developed in the UK and only available in English for some years. From the perspective of (health) economists concerned with economic evaluations, a good outcome measure should possess three main characteristics [2]. First, it should be comparable among diseases and interventions to allow for interpretation in a comparative way for resource allocation purposes. The capability instruments identified in this literature review were developed for specific population groups; hence, a comparison is currently challenging without a standard application of, for instance, the CALYs framework. Second, the instruments should have a scale with interval properties. All instruments provide a summary score; however, only a few are anchored and therefore have interval properties. The ICECAP scores are anchored on no capability and full capability, and the ASCOT scales are anchored on death and full capability. Finally, most economists are looking for an outcome measure for economic evaluation that reflects preferences, either of individual patients or the general public. Instruments with tariffs derived from the general population (ASCOT, ICECAP-A and ICECAP-SCM) or the relevant subpopulation (ICECAP-O) possess this characteristic. On the other hand, reducing capabilities information only to a single, preference-based index value on a scale of 0–1 may limit the actionable policy relevance of the information [39]. The two approaches, however, are not mutually exclusive and more research is needed about the relative values of different capabilities and their variance according to population specifics (e.g. age, disease experience, culture). More information about the weights people allocate to the attributes and levels of capability instruments would be needed to improve our understanding of the relative value of individual capability domains and dimensions.

Major limitations of this study design include that the search was limited to English and German. Next, this review only assessed instruments and studies reported in the literature, and a thorough grey literature search could not be conducted due to difficulties with the search term capability. In terms of grey literature, only dedicated websites of capability instruments were reviewed for relevant information. This resulted in some limitations, for instance, some cost-effectiveness components of studies that have used ASCOT have not been written up as journal articles and fell therefore outside the findings of this review [118, 119]. Furthermore, ongoing research and developments could not be included which could be important in such a dynamically moving area. For example, we found information about ongoing economic evaluations [41, 42, 114] with the identified instruments where results expected to be published soon, additional capability instruments might have been used in unpublished economic evaluations, or some are currently under development. There is a potential need to update this literature review in the future to gather information from this rapidly growing body of literature about the potential development of additional capability measures, the further validation of existing ones, the empirical use of capability measures in economic evaluations, and the lessons learned from these applications.

## Conclusion

There has been an increasing interest in the application of the capability-based approach in economic evaluations of health-related interventions. Different instruments are available and the choice between them should be based on both the research question and the characteristics of the instruments. Further research should focus on the comparison of the existing capability instruments and examining the correlation across capability measures. This would help future researchers in choosing the most suitable capability instrument for their study and provide further information for instrument developers.

## Appendix 1: search strategy

### Embase and Medline via Embase.com

((‘economic evaluation’/exp OR ‘economic*’:ti,ab,kw OR ‘cost-effective*’:ti,ab,kw OR ‘cost-utility’:ti,ab,kw OR ‘cost-benefit’:ti,ab,kw) AND ((‘ascot’:ti,ab,kw OR ‘icecap’:ti,ab,kw OR ‘oxcap-mh’:ti,ab,kw) OR (capabilit* NEXT/2 (perspective OR approach)):ti,ab,kw)) AND (2000:py OR 2001:py OR 2002:py OR 2003:py OR 2004:py OR 2005:py OR 2006:py OR 2007:py OR 2008:py OR 2009:py OR 2010:py OR 2011:py OR 2012:py OR 2013:py OR 2014:py OR 2015:py OR 2016:py OR 2017:py OR 2018:py OR 2019:py) **(*****182 results*****)**.

### Web of science

TS = (“economic evaluation”) OR TI = (“economic evaluation”) OR TS = (“economic assessment”) OR TI = (“economic assessment”) OR TS = (cost-effectiveness) OR TI = (cost-effectiveness) OR TS = (cost-utility) OR TI = (cost-utility) OR TS = (cost-benefit) OR TI = (cost-benefit) AND TS = (“capabilit* approach”) OR TI = (“capabilit* approach”) OR TS = (“capabilit* perspective”) OR TI = (“capabilit* perspective”) OR TS = (ascot) OR TI = (ascot) OR TS = (icecap*) OR TI = (icecap*) OR TS = (oxcap-mh) OR TI = (oxcap-mh)

*Limitations*: Last 20 years

Indexes = SCI-EXPANDED, SSCI, A&HCI, CPCI-S, CPCI-SSH, ESCI (***90 results***)

### Psychinfo

((capabilit* or ascot or oxcap-mh or icecap).ab. or (capabilit* or ascot or oxcap-mh or icecap).ti.) and ((cost-effectiveness or cost-utility or cost-benefit or economic evaluation or economic assessment).ti. or (cost-effectiveness or cost-utility or cost-benefit or economic evaluation or economic assessment).ab.)

*Limitations*: Last 20 years (***82 results***)

### Scopus

TITLE-ABS-KEY ((“capabilit* approach”) OR (“capabilit* perspective”) OR (ascot) OR (oxcap-mh) OR (icecap)) AND TITLE-ABS-KEY ((economic AND evaluation) OR (economic AND assessment) OR cost-effectiveness OR cost-utility OR cost-benefit)

*Limitations*: English, German, last 20 years (***174 results***)

## Appendix 2: data extraction

The final list of extracted data in case of applied papers included

- First author,
- Year of publication (1999, …, 2018),
- Country of study, Disease area,
- Type of intervention,
- Population under investigation,
- Aim of study (to assess cost-effectiveness, to …),
- Type of economic evaluation (cost-minimisation analysis, cost-consequence analysis, cost-effectiveness analysis, cost-utility analysis, cost–benefit analysis, not applicable),
- Perspective of study (healthcare service, societal, other),
- Capability instrument used (multiple choice between: ICECAP-A, ICECAP-O, OxCAP-MH, ASCOT, Other),
- Time points of measurement (pre-study, baseline, post-study, 1 month, …, 12 months, beyond 12 months),
- Other instruments used,
- Methods to address missing data,
- Presentation of results, e.g. cost/CALYs, p value of capability instrument (less than 0.05, greater than 0.05), Comparison of results to QALYs (lower, nearly identical, higher),
- Comparison of incremental cost-effectiveness ratios (difference in costs per extra year to gain well-being, expressed in EUR),
- Use of capability data in economic modelling (yes, no),
- Recommendation to apply capability in future economic evaluations (yes, no),
- Further comments on the capability instrument.

Data extraction in case of methods papers included the

- First author,
- Year of publication (2009, …, 2018),
- Type of study (Comparison of questionnaires; Development of questionnaires; Methods to incorporate CA to economic evaluation; Theoretical background of CA),
- Aim of study,
- Capability instrument in question (multiple choice between: ICECAP-A, ICECAP-O, OxCAP-MH, ASCOT, Other),
- Recommendation to apply capability in future economic evaluations (yes, no),
- Further comments on the capability instrument.

## Appendix 3

See Table 8.

**Table 8:** List of included papers

Author	Year	References	Category	Instrument(s)
Al-Janabi	2012	[53]	Development	ICECAP-A
Al-Janabi	2015	[77]	Validation	ICECAP-A
Al-Janabi	2013	[33]	Validation	ICECAP-A
Al-Janabi	2013	[82]	Validation	ICECAP-A
Bailey	2016	[29]	Validation	ICECAP-SCM
Barnes	2016	[44]	Empirical	ICECAP-A
Baumgardt	2018	[103]	Validation	OxCAP-MH
Botes	2018	[60]	Development	CAF
Botes	2018	[115]	Validation	CAF
Bray	2017	[111]	Empirical	ICECAP-A
Burns	2016	[45]	Empirical	OxCAP-MH
Chen	2018	[20]	Validation	ICECAP-A
Coast	2008	[88]	Valuation	ICECAP-O
Coast	2016	[100]	Valuation	ICECAP-SCM
Coast	2008	[40]	Validation	ICECAP-O
Coast	2018	[83]	Validation	ICECAP-A, ICECAP-SCM
Comans	2012	[97]	Validation	ICECAP-O
Couzner	2012	[22]	Comparison	ICECAP-O
Couzner	2013	[36]	Validation	ICECAP-O
Davis	2013	[25]	Comparison	ICECAP-O
Davis	2016	[30]	Validation	ICECAP-O
Davis	2017	[35]	Validation	ICECAP-O
Engel	2018	[78]	Validation	ICECAP-A
Engel	2018	[79]	Validation	ICECAP-A
Engel	2016	[89]	Validation	ICECAP-O
Engel	2017	[24]	Comparison	ICECAP-A
Flynn	2015	[85]	Valuation	ICECAP-A
Forder	2011	[68]	Validation	ASCOT
Franklin	2018	[26]	Comparison	ICECAP-O
Goranitis	2016	[34]	Comparison	ICECAP-A
Goranitis	2017	[112]	Empirical	ICECAP-A
Goranitis	2016	[23]	Validation	ICECAP-A
Greco	2018	[102]	Validation	low-income Q
Greco	2015	[64]	Development	low-income Q
Grewal	2006	[54]	Development	ICECAP-O
Hackert	2017	[21]	Comparison	ASCOT, ICECAP-O
Hackert	2019	[90]	Validation	ICECAP-O
Handels	2018	[109]	Translation	ICECAP-O
Henderson	2013	[113]	Empirical	ICECAP-O
Horder	2016	[86]	Validation	ICECAP-O
Horwood	2014	[91]	Validation	ICECAP-O
Huynh	2017	[101]	Valuation	ICECAP-SCM
Jones	2017	[32]	Validation	ICECAP-A
Kaambwa	2019	[69]	Validation	ASCOT
Karimi	2016	[49]	Incorporation	General
Keeley	2013	[80]	Validation	ICECAP-A
Keeley	2015	[81]	Validation	ICECAP-A
Keeley	2016	[27]	Comparison	ICECAP-A
Khan	2018	[116]	Validation	ICECAP-A
Kinghorn	2015	[65]	Development	Pain Q
Łaszewska	2019	[17]	Comparison	OxCAP-MH
Linton	2018	[108]	Validation	ICECAP-A
Looman	2014	[98]	Validation	ICECAP-O
Lorgelly	2015	[63]	Development	OCAP-18
Makai	2014	[6]	Validation	ASCOT, ICECAP-O
Makai	2015	[31]	Empirical	ICECAP-O
Makai	2012	[92]	Validation	ICECAP-O
Makai	2014	[18]	Validation	ICECAP-O
Malley	2012	[70]	Validation	ASCOT
Mansdotter	2017	[47]	Incorporation	General
Milte	2014	[71]	Comparison	ASCOT
Milte	2018	[93]	Validation	ICECAP-O
Mitchell	2017	[13]	Incorporation	General
Mitchell	2015	[37]	Validation	ICECAP-A
Mitchell	2013	[94]	Comparison	ICECAP-O
Mitchell	2015	[48]	Incorporation	General
Mitchell	2017	[38]	Comparison	ICECAP-A
Netten	2012	[52]	Development	ASCOT
Parker	2019	[43]	Empirical	ICECAP-A
Parsons	2014	[95]	Validation	ICECAP-O
Patty	2018	[46]	Empirical	ICECAP-O
Peak	2018	[84]	Validation	ICECAP-A
Rand	2017	[72]	Comparison	ASCOT
Rand	2012	[58]	Development	ASCOT-proxy
Ratcliffe	2013	[99]	Validation	ICECAP-O
Sacchetto	2016	[56]	Development	ACQ‐CMH‐104
Sacchetto	2018	[66]	Validation	ACQ‐CMH‐104
Sarabia-Cobo	2017	[87]	Comparison	ICECAP-O
Shiroiwa	2018	[105]	Validation	ASCOT
Simon	Unpublished	[114]	Empirical	OxCAP-MH
Simon	2018	[117]	Translation	OxCAP-MH
Simon	2013	[39]	Development	OxCAP-MH
Stevens	2018	[73]	Comparison	ASCOT
Sutton	2014	[62]	Development	ICECAP-SCM
Tang	2018	[107]	Comparison	ICECAP-A
Towers	2015	[75]	Validation	ASCOT
Towers	2016	[67]	Validation	ASCOT
Turnpenny	2018	[57]	Development	ASCOT Easy Read
Van Leeuwen	2015	[74]	Comparison	ASCOT, ICECAP-O
Van Leeuwen	2015	[106]	Validation	ASCOT
Van Leeuwen	2014	[104]	Validation	ASCOT
Van Leeuwen	2015	[28]	Validation	ASCOT, ICECAP-O
Vergunst	2017	[19]	Comparison	OxCAP-MH
Williams	2016	[42]	Empirical	ICECAP-O
Xin	2017	[96]	Comparison	ICECAP-O

## Appendix 4

See Table 9.

**Table 9:** Correlations reported in the included studies

Capabilities instrument	Compared with (long name)	Compared with (short name)	Country	Population	Number of informants	Measurement of correlation	Value of correlation	Reference
ACQ‐CMH‐104	WHOQOL‐Bref	WHOQOL‐Bref	Portugal	Psychiatric patients participating in community mental health organisations	129	Pearson coefficient	0.60	[66]
ACQ‐CMH‐104	RAS-P	Recovery assessment scale	Portugal	Psychiatric patients participating in community mental health organisations	92	Pearson coefficient	0.46	[66]
ASCOT	EQ-5D-3L	EQ-5D-3L	UK	Day care for older people	224	Spearman Rank	0.47	[68]
ASCOT	ICECAP-O	ICECAP-O	UK	Older social care users	205	Spearman Rank	0.81	[21]
ASCOT	EQ-5D-5L	EQ-5D-5L	UK	Older social care users	205	Spearman Rank	0.63	[21]
ASCOT	EQ-5D-VAS	EQ-5D-VAS	UK	Older social care users	205	Spearman Rank	0.64	[21]
ASCOT	Barthel Index	Barthel Index	UK	Older social care users	205	Spearman Rank	0.45	[21]
ASCOT	GDS-15 (negative correlation)	GDS-15*	UK	Older social care users	205	Spearman Rank	0.69	[21]
ASCOT	OPQOL-13	OPQOL-13	UK	Older social care users	205	Spearman Rank	0.76	[21]
ASCOT	SWLS	SWLS	UK	Older social care users	205	Spearman Rank	0.74	[21]
ASCOT	Cantril’s Ladder	Cantril’s Ladder	UK	Older social care users	205	Spearman Rank	0.66	[21]
ASCOT	Older People’s Quality-of-Life brief questionnaire	OPQoL-Brief	Australia	Community-dwelling older people receiving aged care services	87	Spearman Rank	0.58	[69]
ASCOT	EQ-5D-3L	EQ-5D-3L	UK	Older people receiving publicly funded home care services	301	Pearson correlation	0.40	[70]
ASCOT	EQ-5D-5L	EQ-5D-5L	Australia	Older adults in a day rehabilitation facility	22	Spearman Rank	0.24	[71]
ASCOT	Brief Older People’s Quality of Life	OPQOL-brief	Australia	Older adults in a day rehabilitation facility	22	Spearman Rank	0.38	[71]
ASCOT	EQ-5D-3L	EQ-5D-3L	UK	Older home care residents	301	Pearson coefficient	0.41	[52]
ASCOT	GHQ-12 (negative correlation)	GHQ-12*	UK	Older home care residents	301	Pearson coefficient	0.58	[52]
ASCOT	Control and autonomy subscale of CASP-12	CASP-12	UK	Older home care residents	301	Pearson coefficient	0.58	[52]
ASCOT	EQ-5D-3L	EQ-5D-3L	UK	General population	200	Gradient	0.98	[73]
ASCOT	EQ-5D-3L	EQ-5D-3L	Netherlands	Frail older adults living at home	190	Spearman Rank	0.41	[74]
ASCOT	ICECAP-O	ICECAP-O	Netherlands	Frail older adults living at home	190	Spearman Rank	0.41	[74]
ASCOT	EQ-5D-3L	EQ-5D-3L	UK	Community-based adult social care service users	748	Spearman Rank	0.37	[72]
ASCOT	ICECAP-O	ICECAP-O	UK	Community-based adult social care service users	748	Spearman Rank	0.67	[72]
ASCOT	ICECAP-A	ICECAP-A	UK	Community-based adult social care service users	748	Spearman Rank	0.62	[72]
ASCOT-Carer	Carer Experience Scale (CES)	CES	UK	Social care recipients	376	Spearman Rank	0.58	[76]
ASCOT-Carer	Carer Strain Index (negative correlation)	CSI	UK	Social care recipients	384	Spearman Rank	− 59	[76]
ASCOT-Carer	EQ-5D-3L	EQ-5D-3L	UK	Social care recipients	382	Spearman Rank	0.34	[76]
ASCOT-Carer	QoL (single item using a 7-point Likert scale)	QoL	UK	Social care recipients	384	Spearman Rank	0.62	[76]
ICECAP-A	Assessment of Quality of Life	AQoL-8D	Australia, Canada, Germany, Norway, UK, USA	Patients with seven chronic conditions and a sample of the ‘healthy’ public	8022	Spearman Rank	0.80	[20]
ICECAP-A	EQ-5D-5L	EQ-5D-5L	Australia, Canada, Germany, Norway, UK, USA	Patients with seven chronic conditions and a sample of the ‘healthy’ public	8022	Spearman Rank	0.60	[20]
ICECAP-A	15D	15D	6 countries (MIC)	Representative healthy cohort and from patients in eight clinical areas	6756	Pearson coefficient (average of correlations among factors)	0.50	[24]
ICECAP-A	AQoL-8D	AQoL-8D	6 countries (MIC)	Representative healthy cohort and from patients in eight clinical areas	6756	Pearson coefficient (average of correlations among factors)	0.31	[24]
ICECAP-A	EQ-5D-5L	EQ-5D-5L	6 countries (MIC)	Representative healthy cohort and from patients in eight clinical areas	6756	Pearson coefficient (average of correlations among factors)	0.49	[24]
ICECAP-A	HUI-3	HUI-3	6 countries (MIC)	Representative healthy cohort and from patients in eight clinical areas	6756	Pearson coefficient (average of correlations among factors)	0.32	[24]
ICECAP-A	SF-6D	SF-6D	6 countries (MIC)	Representative healthy cohort and from patients in eight clinical areas	6756	Pearson coefficient (average of correlations among factors)	0.47	[24]
ICECAP-A	HUI-3	HUI-3	Australia, Canada, Germany, Norway, UK, and USA	Individuals with self-reported depression	917	*R*^2^	0.46	[79]
ICECAP-A	SF-6D	SF-6D	Australia, Canada, Germany, Norway, UK, and USA	Individuals with self-reported depression	917	*R*^2^	0.36	[79]
ICECAP-A	15D	15D	Australia, Canada, Germany, Norway, UK, and USA	Individuals with self-reported depression	917	*R*^2^	0.42	[79]
ICECAP-A	Assessment of Quality-of-Life Multi-Attribute Utility Instrument	AQoL-8D	Australia, Canada, Germany, Norway, UK, and USA	Individuals with self-reported depression	917	*R*^2^	0.58	[79]
ICECAP-A	EQ-5D-5L	EQ-5D-5L	Australia, Canada, Germany, Norway, UK, and USA	Individuals with self-reported depression	917	*R*^2^	0.34	[79]
ICECAP-A	EQ-5D-5L	EQ-5D-5L	Canada	Patients with Spinal Cord Injury	364	Path analysis	0.37	[78]
ICECAP-A	Assessment of Quality-of-Life Multi-Attribute Utility Instrument	AQoL-8D	Canada	Patients with Spinal Cord Injury	364	Path analysis	0.54	[78]
ICECAP-A	Leeds Dependence Questionnaire (negative correlation)	LDQ*	UK	Individuals receiving opiate substitution treatment for more than 12 months	83	Pearson coefficient	0.48	[34]
ICECAP-A	Social Satisfaction Questionnaire	SSQ	UK	Individuals receiving opiate substitution treatment for more than 12 months	83	Pearson coefficient	0.43	[34]
ICECAP-A	EQ-5D-3L	EQ-5D-3L	UK	Women with lower urinary tract symptoms	478	Pearson coefficient	0.53	[23]
ICECAP-A	EQ-5D-3L	EQ-5D-3L	UK	Knee pain patients in primary care	500	Spearman Rank	0.49	[27]
ICECAP-A	36-Item Short Form Health Survey	SF-36	Australia, Canada, Germany, Norway, UK, USA	Patients with seven chronic conditions and a sample of the ‘healthy’ public	8022	*R*^2^	0.57	[116]
ICECAP-A	36-Item Short Form Health Survey	AQoL-8D	Australia, Canada, Germany, Norway, UK, USA	Patients with seven chronic conditions and a sample of the ‘healthy’ public	8022	*R*^2^	0.71	[116]
ICECAP-A	EQ-5D-5L	EQ-5D-5L	Germany	Healthy Samples and Seven Health Condition Groups	1212	Pearson coefficient	0.62	[108]
ICECAP-A	SWLS	SWLS	Germany	Healthy Samples and Seven Health Condition Groups	1212	Pearson coefficient	0.66	[108]
ICECAP-A	SF-6D	SF-6D	Germany	Healthy Samples and Seven Health Condition Groups	1212	Pearson coefficient	0.64	[108]
ICECAP-A	Depression, Anxiety and Stress Scale	DASS-D	4 English speaking countries of MIC	Individuals with depression	617	*R*^2^	?	[38]
ICECAP-A	Kessler Psychological Distress Scale	K10	4 English speaking countries of MIC	Individuals with depression	617	*R*^2^	?	[38]
ICECAP-A	EQ-5D-3L	EQ-5D-3L	China	General population	975	Polychoric correlation coefficient	0.45	[107]
ICECAP-O	EQ-5D-3L	EQ-5D-3L	UK	General population aged 65 and over	315	Chi-squared tests	0.42 (Attachment), 0.008** (Security), < 0.001** (Role), < 0.001** (Enjoyment), < 0.001** (Control)	[40]
ICECAP-O	EQ-5D	EQ-5D-3L	Australia	Patients from an outpatient day rehabilitation unit	80	Spearman Rank	0.44	[22]
ICECAP-O	CTM-3	CTM-3	Australia	Patients from an outpatient day rehabilitation unit	82	Spearman Rank	0.23	[22]
ICECAP-O	EQ-5D	EQ-5D-3L	Canada	Participants visiting the Vancouver Falls Prevention Clinic	215	Spearman Rank	0.47	[25]
ICECAP-O	EQ-5D-3L	EQ-5D-3L	UK	Aged over 65 years, requiring a hospital visit and/or care home resident, and recruited to one of 3 studies forming the Medical Crisis in Older People (MCOP) programme	584	*R*^2^	0.35	[26]
ICECAP-O	EQ-5D-5L	EQ-5D-5L	UK	Older social care users	207	Spearman Rank	0.68	[21]
ICECAP-O	EQ-5D-VAS	EQ-5D-VAS	UK	Older social care users	208	Spearman Rank	0.66	[21]
ICECAP-O	Barthel Index	Barthel Index	UK	Older social care users	209	Spearman Rank	0.49	[21]
ICECAP-O	GDS-15 (negative correlation)	GDS-15*	UK	Older social care users	210	Spearman Rank	0.73	[21]
ICECAP-O	OPQOL-13	OPQOL-13	UK	Older social care users	211	Spearman Rank	0.80	[21]
ICECAP-O	SWLS	SWLS	UK	Older social care users	212	Spearman Rank	0.82	[21]
ICECAP-O	Cantril’s Ladder	Cantril’s Ladder	UK	Older social care users	213	Spearman Rank	0.74	[21]
ICECAP-O	EQ-5D-5L	EQ-5D-5L	UK	People aged 70 and older	516	Spearman Rank	0.63	[90]
ICECAP-O	Barthel Index	Barthel Index	Germany	Nursing Home Residents with Dementia	95	Pearson coefficient	0.72	[18]
ICECAP-O	EQ-5D-3L	EQ-5D-3L	Germany	Nursing Home Residents with Dementia	95	Pearson coefficient	0.69	[18]
ICECAP-O	ADRQL	ADRQL	Germany	Nursing Home Residents with Dementia	95	Pearson coefficient	0.53	[18]
ICECAP-O	EQ-5D-3L	EQ-5D-3L	Australia	Older people following surgery for hip fracture	87	Spearman Rank	0.53	[93]
ICECAP-O	Western Ontario and McMaster Universities	WOMAC	UK	Osteoarthritis patients requiring joint replacement	105	*R*^2^	0.40	[94]
ICECAP-O	EQ-5D-3L	EQ-5D-3L	UK	Participants aged 65 years and over with an intracapsular fracture of the hip	113	Pearson coefficient	0.34	[95]
ICECAP-O	Oxford Hip Score	OHS	UK	Participants aged 65 years and over with an intracapsular fracture of the hip	113	Pearson coefficient	0.38	[95]
ICECAP-O	Barthel Index measure of activities of daily living	Barthel Index	Spain	Nursing professionals serving as proxy respondents for dementia patients	217	Not reported	0.68	[87]
ICECAP-O	Alzheimer’s Disease-Related Quality of Life	ADRQL	Spain	Nursing professionals serving as proxy respondents for dementia patients	217	Not reported	0.61	[87]
ICECAP-O	EQ-5D extended with a cognitive dimension	EQ-5D + C	Spain	Nursing professionals serving as proxy respondents for dementia patients	217	Not reported	0.62	[87]
ICECAP-O	EQ-5D-3L	EQ-5D-3L	Netherlands	Frail older adults living at home	190	Spearman Rank	0.63	[74]
ICECAP-O	Parkinson’s specific QoL	PDQ-39	?	People with Parkinson’s	1023	Not reported	0.53	[96]
ICECAP-O family version	EQ-5D family version	EQ-5D family version	Netherlands	Nursing professionals of psycho-geriatric elderly	96	Pearson coefficient	0.57	[92]
ICECAP-O family version	EQ-VAS family version	EQ-VAS family version	Netherlands	Family members of psycho-geriatric elderly	68	Pearson coefficient	0.43	[92]
ICECAP-O nursing version	EQ-5D nursing version	EQ-5D nursing version	Netherlands	Nursing professionals of psycho-geriatric elderly	96	Pearson coefficient	0.48	[92]
ICECAP-O nursing version	EQ-VAS nursing version	EQ-VAS nursing version	Netherlands	Family members of psycho-geriatric elderly	68	Pearson coefficient	0.55	[92]
OxCAP-MH	EQ-5D-index UK	EQ-5D-index UK	Austria	Patients in socio-psychiatric services	159	Spearman Rank	0.67	[17]
OxCAP-MH	EQ-5D-index DE	EQ-5D-index DE	Austria	Patients in socio-psychiatric services	160	Spearman Rank	0.66	[17]
OxCAP-MH	EQ-5D VAS	EQ-5D VAS	Austria	Patients in socio-psychiatric services	161	Spearman Rank	0.58	[17]
OxCAP-MH	BSI-18	BSI-18	Austria	Patients in socio-psychiatric services	162	Spearman Rank	− 67	[17]
OxCAP-MH	WHOQOL-BREF Physical health	WHOQOL-BREF Physical health	Austria	Patients in socio-psychiatric services	163	Spearman Rank	0.69	[17]
OxCAP-MH	WHOQOL-BREF Psychological	WHOQOL-BREF Psychological	Austria	Patients in socio-psychiatric services	164	Spearman Rank	0.75	[17]
OxCAP-MH	WHOQOL-BREF Social relationships	WHOQOL-BREF Social relationships	Austria	Patients in socio-psychiatric services	165	Spearman Rank	0.50	[17]
OxCAP-MH	WHOQOL-BREF Environment	WHOQOL-BREF Environment	Austria	Patients in socio-psychiatric services	166	Spearman Rank	0.69	[17]
OxCAP-MH	Mini-ICF-APP	Mini-ICF-APP	Austria	Patients in socio-psychiatric services	167	Spearman Rank	− 0.47	[17]
OxCAP-MH	Global Assessment of Functioning	GAF	Austria	Patients in socio-psychiatric services	168	Spearman Rank	0.35	[17]
OxCAP-MH	EQ-5D-3L Utility	EQ-5D-3L	UK	Patients with psychosis	172	Pearson coefficient	0.45	[19]
OxCAP-MH	EuroQol Visual Analogue Scale	EQ-5D-VAS	UK	Patients with psychosis	172	Pearson coefficient	0.52	[19]
OxCAP-MH	Brief Psychiatric Rating Scale (negative correlation)	BPRS*	UK	Patients with psychosis	172	Pearson coefficient	0.41	[19]
OxCAP-MH	Global Assessment of Functioning	GAF	UK	Patients with psychosis	172	Pearson coefficient	0.24	[19]
OxCAP-MH	Objective Social Outcomes Index	SIX	UK	Patients with psychosis	172	Pearson coefficient	0.12	[19]
Women’s Capabilities Index	WHOQOL-Bref	WHOQOL-Bref	Malawi	Women from Mchinji, Malawi	20	Pearson correlation	0.62	[64]

## Appendix 5

See Table 10.

**Table 10:** Abbreviations of health-related instruments

Short form	Full name of instrument
15D	15D
SF-36	36-Item Short Form Health Survey
ADRQL	Alzheimer’s Disease-Related Quality of Life
AQoL-8D	Assessment of Quality-of-Life Multi-Attribute Utility Instrument
Barthel Index	Barthel Index measure of activities of daily living (ADL)
OPQOL-brief	brief Older People’s Quality of Life
BPRS	Brief Psychiatric Rating Scale
BSI-18	brief symptom inventory 18
Cantril’s Ladder	Cantril’s Ladder
CES	Carer Experience Scale
CSI	Carer Strain Index
CASP-12	Control and autonomy subscale of CASP-12
CTM-3	Care Transitions Measure
DASS-D	Depression, Anxiety and Stress Scale (DASS-D of DASS-21)
EQ-5D + C	EQ-5D extended with a cognitive dimension
EQ-5D-VAS	EuroQol Visual Analogue Scale
GDS-15	15-item Geriatric Depression Scale
GHQ-12	12-item General Health Questionnaire
GAF	Global Assessment of Functioning
HUI-3	Health Utilities Index Mark 3
K10	Kessler Psychological Distress Scale
LDQ	Leeds Dependence Questionnaire
Mini-ICF-APP	Mini-ICF-APP Social Functioning Scale
SIX	Objective Social Outcomes Index
OPQoL-Brief	Older People’s Quality-of-Life brief questionnaire (13 items)
OHS	Oxford Hip Score
PDQ-39	Parkinson’s specific Quality of Life
RAS-P	Recovery Assessment Scale
SF-6D	Short Form Six Dimension
SSQ	Social Satisfaction Questionnaire
SWLS	Satisfaction with Life Scale
WOMAC	Western Ontario and McMaster Universities
WHOQOL-Bref	World Health Organization Quality-of-Life Instruments - abbreviated version

## Appendix 6

See Table 11.

**Table 11:** Details of applied evaluations

Author, Year	Country	Disease	Intervention	Population	Perspective	Capability measure	Time points	Missing data
Barnes, 2016	UK	Schizophrenia	Citalopram (ACTIONS trial)	Adult patients	Societal	ICECAP-A	Baseline; 12–36–48 weeks	Multiple imputation
Bray, 2017	UK	Visual impairment	Portable electronic vision enhancement system (compared with optical low vision aids)	Adult patients	Societal	ICECAP-A	Baseline; 2 months; 4 months	Not reported
Burns, 2016	UK	Psychosis	Community treatment orders	Adult patients	Health and social care	OxCAP-MH	Baseline; 6 months; 12 months	Multiple imputation
Goranitis, 2017	UK	Drug addiction	2 Psychological interventions relative to usual care	Treatment resistant adult addicts	Health and social care	ICECAP-A	Baseline; 12 months	Chained equations with predictive mean matching
Henderson, 2013	UK	Heart failure, chronic obstructive pulmonary disease, or diabetes	Community-based telehealth (Whole Systems Demonstrator)	People with a long-term condition	Societal	ICECAP-O	Baseline; 12 months	Multiple imputation
Makai, 2014	Netherlands	Health decline in the elderly	Walcheren integrated care model	Frail elderly	Societal	ICECAP-O	Baseline, 3 months	Not reported
Parker, 2019	UK	Diabetic plantar ulceration	Traditional vs. digital foot orthoses	Adult patients	Healthcare provider	ICECAP-A	Baseline; 6 months	Not reported
Patty, 2018	Netherlands	Visual impairment	ICT training	Adult patients	Societal	ICECAP-O	3 months; post-intervention; pre-study	Not reported
Simon, unpublished	UK	Schizophrenia or schizoaffective disorder and depression	Positive Memory Training (PoMeT)	Adult patients	(1) Healthcare, (2) Health and social care, (3) Broader societal	ICECAP-A and OxCAP-MH	Baseline, 3, 6 and 9 months	Stepwise approach
Williams, 2016	UK	Hip fracture	Multidisciplinary rehabilitation package following hip fracture	Older adults (aged ≥ 65)	Healthcare provider	ICECAP-O	Baseline, 3 months	Not reported

## References

[1] Drummond MF, Sculpher MJ, Torrance GW, O’Brien BJ, Stoddart GL (2005). Standard Methods for the economic evaluation of health care programme.

[2] Fox-Rushby JCJ, Fox-Rushby JCJ (2008). Approaches to measuring health and life. Economic evaluation.

[3] Nord E (2018). Beyond QALYs: Multi-criteria based estimation of maximum willingness to pay for health technologies. The European Journal of Health Economics.

[4] Greco G, Lorgelly P, Yamabhai I (2016). Outcomes in economic evaluations of public health interventions in low- and middle-income countries: Health capabilities and subjective wellbeing. Health Economics.

[5] Makai P, Brouwer WBF, Koopmanschap MA, Stolk EA, Nieboer AP (2014). Quality of life instruments for economic evaluations in health and social care for older people: A systematic review. Social Science and Medicine.

[6] Al-Janabi H, Flynn TN, Coast J (2011). QALYs and carers. PharmacoEconomics.

[7] Sen A (1985). Commodities and capabilities.

[8] Coast J, Flynn T, Sutton E, Al-Janabi H, Vosper J, Lavender S (2008). Investigating choice experiments for preferences of older people (ICEPOP): Evaluative spaces in health economics. Journal of Health Services Research and Policy.

[9] Nederland, Z. (2016). Guideline for economic evaluations in healthcare. Retrieved from https://english.zorginstituutnederland.nl/publications/reports/2016/06/16/guideline-for-economic-evaluations-in-healthcare.

[10] NICE. (2017). Developing NICE guidelines: The manual 2014 (April 2017 update)—Incorporating economic evaluation. [PMG20]: National Institute for Health and Clinical Excellence.

[11] Proud L, McLoughlin C, Kinghorn P (2019). ICECAP-O, the current state of play: A systematic review of studies reporting the psychometric properties and use of the instrument over the decade since its publication. Quality of Life Research.

[12] Hartley RJ, Keen EM, Large JA, Tedd LA, Hartley RJ (1990). Search strategies. Online searching: Principles and practice.

[13] Mitchell PM, Roberts TE, Barton PM, Coast J (2017). Applications of the capability approach in the health field: A literature review. Social Indicators Research.

[14] Anand P, Wailoo A (2000). Utilities versus rights to publicly provided goods: Arguments and evidence from health care rationing. Economica.

[15] Mokkink LB, Terwee CB, Patrick DL, Alonso J, Stratford PW, Knol DL (2010). The COSMIN checklist for assessing the methodological quality of studies on measurement properties of health status measurement instruments: An international Delphi study. Quality of Life Research.

[16] Stinson JN, Kavanagh T, Yamada J, Gill N, Stevens B (2006). Systematic review of the psychometric properties, interpretability and feasibility of self-report pain intensity measures for use in clinical trials in children and adolescents. Pain.

[17] Laszewska A, Schwab M, Leutner E, Oberrauter M, Spiel G, Simon J (2019). Measuring broader wellbeing in mental health services: Validity of the German language OxCAP-MH capability instrument. Quality of Life Research.

[18] Makai P, Beckebans F, Van Exel J, Brouwer WBF (2014). Quality of life of nursing home residents with dementia: Validation of the German version of the ICECAP-O. PLoS ONE.

[19] Vergunst F, Jenkinson C, Burns T, Anand P, Gray A, Rugkåsa J (2017). Psychometric validation of a multi-dimensional capability instrument for outcome measurement in mental health research (OxCAP-MH). Health and Quality of Life Outcomes.

[20] Chen G, Ratcliffe J, Kaambwa B, McCaffrey N, Richardson J (2018). Empirical comparison between capability and two health-related quality of life measures. Social Indicators Research.

[21] Hackert MQN, Exel JV, Brouwer WBF (2017). Valid outcome measures in care for older people: Comparing the ASCOT and the ICECAP-O. Value in Health.

[22] Couzner L, Ratcliffe J, Crotty M (2012). The relationship between quality of life, health and care transition: An empirical comparison in an older post-acute population. Health and Quality of Life Outcomes.

[23] Goranitis I, Coast J, Al-Janabi H, Latthe P, Roberts TE (2016). The validity and responsiveness of the ICECAP-A capability-well-being measure in women with irritative lower urinary tract symptoms. Quality of Life Research.

[24] Engel L, Mortimer D, Bryan S, Lear SA, Whitehurst DGT (2017). An investigation of the overlap between the ICECAP-A and five preference-based health-related quality of life instruments. PharmacoEconomics.

[25] Davis JC, Liu-Ambrose T, Richardson CG, Bryan S (2013). A comparison of the ICECAP-O with EQ-5D in a falls prevention clinical setting: Are they complements or substitutes?. Quality of Life Research.

[26] Franklin M, Payne K, Elliott RA (2018). Quantifying the relationship between capability and health in older people: Can’t Map Won’t Map. Medical Decision Making.

[27] Keeley T, Coast J, Nicholls E, Foster NE, Jowett S, Al-Janabi H (2016). An analysis of the complementarity of ICECAP-A and EQ-5D-3 L in an adult population of patients with knee pain. Health and Quality of Life Outcomes.

[28] van Leeuwen KM, Jansen AP, Muntinga ME, Bosmans JE, Westerman MJ, van Tulder MW (2015). Exploration of the content validity and feasibility of the EQ-5D-3L, ICECAP-O and ASCOT in older adults. BMC Health Services Research.

[29] Bailey C, Kinghorn P, Orlando R, Armour K, Perry R, Jones L (2016). The ICECAP-SCM tells you more about what I’m going through’: A think-aloud study measuring quality of life among patients receiving supportive and palliative care. Palliative Medicine.

[30] Davis JC, Hsiung GY, Bryan S, Jacova C, Jacova P, Munkacsy M (2016). Agreement between patient and proxy assessments of quality of life among older adults with vascular cognitive impairment using the EQ-5D-3L and ICECAP-O. PLoS ONE.

[31] Makai P, Looman W, Adang E, Melis R, Stolk E, Fabbricotti I (2015). Cost-effectiveness of integrated care in frail elderly using the ICECAP-O and EQ-5D: Does choice of instrument matter?. European Journal of Health Economics.

[32] Jones CJ, Payne K, Gannon B, Verstappen S (2017). Exploring the impact of health status and well-being of people with inflammatory arthritis on presenteeism in the workplace: A qualitative study. Annals of the Rheumatic Diseases.

[33] Al-Janabi H, Peters TJ, Brazier J, Bryan S, Flynn TN, Clemens S (2013). An investigation of the construct validity of the ICECAP-A capability measure. Quality of Life Research: An International Journal of Quality of Life Aspects of Treatment, Care and Rehabilitation.

[34] Goranitis I, Coast J, Day E, Copello A, Freemantle N, Seddon J (2016). Measuring Health and Broader Well-Being Benefits in the Context of Opiate Dependence: The Psychometric Performance of the ICECAP-A and the EQ-5D-5L. Value in Health.

[35] Davis JC, Best JR, Dian L, Khan KM, Hsu CL, Chan W (2017). Are the EQ-5D-3L and the ICECAP-O responsive among older adults with impaired mobility? Evidence from the Vancouver Falls Prevention Cohort Study. Quality of Life Research.

[36] Couzner L, Crotty M, Norman R, Ratcliffe J (2013). A comparison of the EQ-5D-3L and CECAP-O in an older post-acute patient population relative to the general population. Applied Health Economics and Health Policy.

[37] Mitchell PM, Al-Janabi H, Richardson J, Iezzi A, Coast J (2015). The relative impacts of disease on health status and capability wellbeing: A multi-country study. PLoS ONE.

[38] Mitchell PM, Al-Janabi H, Byford S, Kuyken W, Richardson J, Iezzi A (2017). Assessing the validity of the ICECAP-A capability measure for adults with depression. BMC Psychiatry.

[39] Simon J, Anand P, Gray A, Rugkasa J, Yeeles K, Burns T (2013). Operationalising the capability approach for outcome measurement in mental health research. Social Science and Medicine.

[40] Coast J, Peters TJ, Natarajan L, Sproston K, Flynn T (2008). An assessment of the construct validity of the descriptive system for the ICECAP capability measure for older people. Quality of Life Research.

[41] Deidda M, Coll-Planas L, Giné-Garriga M, Guerra-Balic M, Roqué Figuls MI, Tully MA (2018). Cost-effectiveness of exercise referral schemes enhanced by self-management strategies to battle sedentary behaviour in older adults: Protocol for an economic evaluation alongside the SITLESS three-armed pragmatic randomised controlled trial. British Medical Journal Open.

[42] Williams NH, Roberts JL, Din NU, Totton N, Charles JM, Hawkes CA (2016). Fracture in the Elderly Multidisciplinary Rehabilitation (FEMuR): A phase II randomised feasibility study of a multidisciplinary rehabilitation package following hip fracture. British Medical Journal Open.

[43] Parker DJ, Nuttall GH, Bray N, Hugill T, Martinez-Santos A, Edwards RT (2019). A randomised controlled trial and cost-consequence analysis of traditional and digital foot orthoses supply chains in a National Health Service setting: Application to feet at risk of diabetic plantar ulceration. Journal of foot and ankle research.

[44] Barnes TRE, Leeson VC, Paton C, Costelloe C, Simon J, Kiss N (2016). Antidepressant controlled trial for negative symptoms in schizophrenia (ACTIONS): A double-blind, placebo-controlled, randomised clinical trial. Health Technology Assessment.

[45] Burns T, R. J., Yeeles K, Catty J, on behalf of the Oxford Mental Health Coercion (OCTET) Programme Group (2016). Coercion in mental health: A trial of the effectiveness of community treatment orders and an investigation of informal coercion in community mental health care. *Programme Grants Applied Research* (Vol. 4). 27997090

[46] Patty NJS, Koopmanschap M, Holtzer-Goor K (2018). A cost-effectiveness study of ICT training among the visually impaired in the Netherlands. BMC ophthalmology.

[47] Månsdotter A, Ekman B, Feldman I, Hagberg L, Hurtig AK, Lindholm L (2017). We propose a novel measure for social welfare and public health: Capability-adjusted life-years, CALYs. Applied Health Economics and Health Policy.

[48] Mitchell PM, Roberts TE, Barton PM, Coast J (2015). Assessing sufficient capability: A new approach to economic evaluation. Social Science and Medicine.

[49] Karimi M, Brazier J, Basarir H (2016). The capability approach: A critical review of its application in health economics. Value in Health.

[50] Lorgelly PK (2015). Choice of outcome measure in an economic evaluation: A potential role for the capability approach. PharmacoEconomics.

[51] Hopkins, W. G. (2002). A new view of statistics. Retrieved February 21, 2019, from, http://www.sportsci.org/resource/stats/effectmag.html.

[52] Netten A, Burge P, Malley J, Potoglou D, Towers AM, Brazier J, Flynn T, Forder J, Wall B (2012). Outcomes of social care for adults: Developing a preference-weighted measure. Health Technology Assessment.

[53] Al-Janabi HF, Flynn TN, Coast J (2012). Development of a self-report measure of capability wellbeing for adults: The ICECAP-A. Quality of Life Research.

[54] Grewal I, Lewis J, Flynn T, Brown J, Bond J, Coast J (2006). Developing attributes for a generic quality of life measure for older people: Preferences or capabilities?. Social Science Medicine.

[55] Coast J, Kinghorn P, Mitchell P (2015). The development of capability measures in health economics: Opportunities challenges and progress. Patient.

[56] Sacchetto B, Aguiar R, Vargas-Moniz MJ, Jorge-Monteiro MF, Neves MJ, Cruz MA (2016). The capabilities questionnaire for the community mental health context (CQ-CMH): A measure inspired by the capabilities approach and constructed through consumer-researcher collaboration. Psychiatric Rehabilitation Journal.

[57] Turnpenny A, Caiels J, Whelton B, Richardson L, Beadle-Brown J, Crowther T (2018). Developing an easy read version of the adult social care outcomes toolkit (ASCOT). Journal of Applied Research in Intellectual Disabilities.

[58] Rand S, Caiels J, Collins G, Forder J (2017). Developing a proxy version of the Adult social care outcome toolkit (ASCOT). Health and Quality of Life Outcomes.

[59] Rand, S., Malley, J., & Netten, A. (2012). Measuring the social care outcomes of informal carers: An interim technical report for the identifying the impact of adult social care (IIASC) Study. In C. a. L. Personal Social Services Research Unit (PSSRU) (Ed.), (Discussion paper ed.).

[60] Botes R, Vermeulen KM, Gerber AM, Ranchor AV, Buskens E (2018). Functioning and quality of life in dutch oldest old with diverse levels of dependency. Patient Preference and Adherence.

[61] Canaway A, Al-Janabi H, Kinghorn P, Bailey C, Coast J (2017). Development of a measure (ICECAP-Close Person Measure) through qualitative methods to capture the benefits of end-of-life care to those close to the dying for use in economic evaluation. Palliative Medicine.

[62] Sutton EJ, Coast J (2014). Development of a supportive care measure for economic evaluation of end-of-life care using qualitative methods. Palliative Medicine.

[63] Lorgelly PK, Lorimer K, Fenwick EAL, Briggs AH, Anand P (2015). Operationalising the capability approach as an outcome measure in public health: The development of the OCAP-18. Social Science and Medicine.

[64] Greco G, Skordis-Worall J, Mkandawire B, Mills A (2015). What is a good life? Selecting capabilities to assess women’s quality of life in rural Malawi. Social Science and Medicine.

[65] Kinghorn P, Robinson A, Smith RD (2015). Developing a capability-based questionnaire for assessing well-being in patients with chronic pain. Social Indicators Research.

[66] Sacchetto B, Ornelas J, Calheiros MM, Shinn M (2018). Adaptation of Nussbaum’s capabilities framework to community mental health: A consumer-based capabilities measure. American Journal of Community Psychology.

[67] Towers AM, Smith N, Palmer S, Welch E, Netten A (2016). The acceptability and feasibility of using the Adult Social Care Outcomes Toolkit (ASCOT) to inform practice in care homes. BMC Health Services Research.

[68] Forder JE, Caiels J (2011). Measuring the outcomes of long-term care. Social Science and Medicine.

[69] Kaambwa B, Gill L, McCaffrey N, Lancsar E, Cameron ID, Crotty M (2015). An empirical comparison of the OPQoL-Brief, EQ-5D-3L and ASCOT in a community dwelling population of older people. Health and Quality of Life Outcomes.

[70] Malley JN, Towers AM, Netten AP, Brazier JE, Forder JE, Flynn T (2012). An assessment of the construct validity of the ASCOT measure of social care-related quality of life with older people. Health and Quality of Life Outcomes.

[71] Milte CM, Walker R, Luszcz MA, Lancsar E, Kaambwa B, Ratcliffe J (2014). How important is health status in defining quality of life for older people? An exploratory study of the views of older South Australians. Applied Health Economics and Health Policy.

[72] Rand S, Malley J, Towers AM, Netten A, Forder J (2017). Validity and test-retest reliability of the self-completion adult social care outcomes toolkit (ASCOT-SCT4) with adults with long-term physical, sensory and mental health conditions in England. Health and Quality of Life Outcomes.

[73] Stevens K, Brazier J, Rowen D (2018). Estimating an exchange rate between the EQ-5D-3L and ASCOT. European Journal of Health Economics.

[74] van Leeuwen KM, Bosmans JE, Jansen APD, Hoogendijk EO, van Tulder MW, van der Horst HE (2015). Comparing measurement properties of the EQ-5D-3L, ICECAP-O, and ASCOT in frail older adults. Value in Health.

[75] Towers AM, Holder J, Smith N, Crowther T, Netten A, Welch E (2015). Adapting the adult social care outcomes toolkit (ASCOT) for use in care home quality monitoring: Conceptual development and testing. BMC Health Services Research.

[76] Rand SE, Malley JN, Netten AP, Forder JE (2015). Factor structure and construct validity of the Adult Social Care Outcomes Toolkit for Carers (ASCOT-Carer). Quality of Life Research.

[77] Al-Janabi H, Flynn TN, Peters TJ, Bryan S, Coast J (2015). Test-retest reliability of capability measurement in the UK general population. Health Economics (United Kingdom).

[78] Engel L, Bryan S, Noonan VK, Whitehurst DGT (2018). Using path analysis to investigate the relationships between standardized instruments that measure health-related quality of life, capability wellbeing and subjective wellbeing: An application in the context of spinal cord injury. Social Science and Medicine.

[79] Engel L, Chen G, Richardson J, Mihalopoulos C (2018). The impact of depression on health-related quality of life and wellbeing: Identifying important dimensions and assessing their inclusion in multi-attribute utility instruments. Quality of Life Research.

[80] Keeley T, Al-Janabi H, Lorgelly P, Coast J (2013). A qualitative assessment of the content validity of the ICECAP-A and EQ-5D-5L and their appropriateness for use in health research. PLoS ONE.

[81] Keeley T, Al-Janabi H, Nicholls E, Foster NE, Jowett S, Coast J (2015). A longitudinal assessment of the responsiveness of the ICECAP-A in a randomised controlled trial of a knee pain intervention. Quality of Life Research.

[82] Al-Janabi H, Keeley T, Mitchell P, Coast J (2013). Can capabilities be self-reported? A think aloud study. Social Science and Medicine.

[83] Coast J, Bailey C, Orlando R, Armour K, Perry R, Jones L (2018). Adaptation, acceptance and adaptive preferences in health and capability well-being measurement amongst those approaching end of life. Patient-Patient Centered Outcomes Research.

[84] Peak J, Goranitis I, Day E, Copello A, Freemantle N, Frew E (2018). Predicting health-related quality of life (EQ-5D-5 L) and capability wellbeing (ICECAP-A) in the context of opiate dependence using routine clinical outcome measures: CORE-OM, LDQ and TOP. Health and Quality of Life Outcomes.

[85] Flynn TN, Huynh E, Peters TJ, Al-Janabi H, Clemens S, Moody A (2015). Scoring the icecap-a capability instrument. Estimation of a UK general population tariff. Health Economics.

[86] Hörder H, Gustafsson S, Rydberg T, Skoog I, Waern M (2016). A cross-cultural adaptation of the ICECAP-O: Test–retest reliability and item relevance in Swedish 70-year-olds. Societies.

[87] Sarabia-Cobo CM, Paras-Bravo P, Amo-Setien FJ, Alconero-Camarero AR, Saenz-Jalon M, Torres-Manrique B (2017). Validation of the Spanish Version of the ICECAP-O for nursing home residents with dementia. PLoS ONE.

[88] Coast J, Flynn TN, Natarajan L, Sproston K, Lewis J, Louviere JJ (2008). Valuing the ICECAP capability index for older people. Social Science and Medicine.

[89] Engel L, Chudyk AM, Ashe MC, McKay HA, Whitehurst DGT, Bryan S (2016). Older adults’ quality of life —exploring the role of the built environment and social cohesion in community-dwelling seniors on low income. Social Science and Medicine.

[90] Hackert MQN, van Exel J, Brouwer WBF (2019). Does the ICECAP-O cover the physical, mental and social functioning of older people in the UK? [Article]. Quality of Life Research.

[91] Horwood J, Sutton E, Coast J (2014). Evaluating the face validity of the ICECAP-O capabilities measure: A “Think Aloud” study with hip and knee arthroplasty patients. Applied Research in Quality of Life.

[92] Makai P, Brouwer WBF, Koopmanschap MA, Nieboer AP (2012). Capabilities and quality of life in Dutch psycho-geriatric nursing homes: An exploratory study using a proxy version of the ICECAP-O. Quality of Life Research.

[93] Milte R, Crotty M, Miller MD, Whitehead C, Ratcliffe J (2018). Quality of life in older adults following a hip fracture: An empirical comparison of the ICECAP-O and the EQ-5D-3 L instruments. Health and Quality of Life Outcomes.

[94] Mitchell PM, Roberts TE, Barton PM, Pollard BS, Coast J (2013). Predicting the ICECAP-O capability index from the WOMAC osteoarthritis index: Is mapping onto capability from condition-specific health status questionnaires feasible?. Medical Decision Making.

[95] Parsons N, Griffin XL, Achten J, Costa ML (2014). Outcome assessment after hip fracture: Is EQ-5D the answer? [Article]. Bone and Joint Research.

[96] Xin Y, Lewsey J, Gray R, Clarke CE, Coast J, Rick C (2017). Too broad to be sensitive? Exploring the responsiveness of the ICECAP-O capability wellbeing measure compared to the EQ-5D-3L to the change of clinical and QoL aspects in people with parkinson’s. Value in Health.

[97] Comans TA, Scuffham PA, Gray L, Peel N (2012). Utility values for use in health care decision making for older frail adults. Value in Health.

[98] Looman WM, Fabbricotti IN, Huijsman R (2014). The short-term effects of an integrated care model for the frail elderly on health, quality of life, health care use and satisfaction with care. International Journal of Integrated Care.

[99] Ratcliffe J, Lester LH, Couzner L, Crotty M (2013). An assessment of the relationship between informal caring and quality of life in older community-dwelling adults - more positives than negatives?. Health and Social Care in the Community.

[100] Coast J, Huynh E, Kinghorn P, Flynn T (2016). Complex valuation: Applying ideas from the complex intervention framework to valuation of a new measure for end-of-life care. PharmacoEconomics.

[101] Huynh E, Coast J, Rose J, Kinghorn P, Flynn T (2017). Values for the ICECAP-Supportive Care Measure (ICECAP-SCM) for use in economic evaluation at end of life. Social Science and Medicine.

[102] Greco G, Skordis-Worrall J, Mills A (2018). Development, validity, and reliability of the women’s capabilities index. Journal of Human Development and Capabilities.

[103] Baumgardt J, Daum M, Von Dem Knesebeck O, Speck A, Röh D (2018). Assess capabilities among chronically mentally ill people: First test results on a draft german version of the OxCAP-MH as Part of the BAESCAP Study. Psychiatrische Praxis.

[104] van Leeuwen KM, Malley J, Bosmans JE, Jansen AP, Ostelo RW, van der Horst HE, Netten A (2014). What can local authorities do to improve the social care-related quality of life of older adults living at home? Evidence from the Adult Social Care Survey. Health and Place.

[105] Shiroiwa T, Moriyama Y, Nakamura-Thomas H, Morikawa M, Fukuda T, Batchelder L (2018). Development of japanese preference weight for the adult social care outcomes toolkit (ASCOT) SCT4. Value in Health.

[106] van Leeuwen KM, Bosmans JE, Jansen APD, Rand SE, Towers AM, Smith N (2015). Dutch translation and cross-cultural validation of the Adult Social Care Outcomes Toolkit (ASCOT). Health and Quality of Life Outcomes.

[107] Tang C, Xiong Y, Wu H, Xu J (2018). Adaptation and assessments of the Chinese version of the ICECAP-A measurement. Health and Quality of Life Outcomes.

[108] Linton MJ, Mitchell PM, Al-Janabi H, Schlander M, Richardson J, Iezzi A (2018). Comparing the german translation of the ICECAP-A capability wellbeing measure to the original english version: Psychometric properties across healthy samples and seven health condition groups. Applied Research in Quality of Life.

[109] Handels RLH, Sköldunger A, Bieber A, Edwards RT, Gonçalves-Pereira M, Hopper L (2018). Quality of life, care resource use, and costs of dementia in 8 European countries in a cross-sectional cohort of the actifcare study. Journal of Alzheimer’s Disease.

[110] Coast J, Bailey C, Canaway A, Kinghorn P, Round J (2016). Measuring and valuing outcomes for care at the end of life: The capability approach. Care at the end of life: An economic perspective.

[111] Bray N, Brand A, Taylor J, Hoare Z, Dickinson C, Edwards RT (2017). Portable electronic vision enhancement systems in comparison with optical magnifiers for near vision activities: An economic evaluation alongside a randomized crossover trial. Acta Ophthalmologica.

[112] Goranitis I, Coast J, Day E, Copello A, Freemantle N, Frew E (2017). Maximizing health or sufficient capability in economic evaluation? A methodological experiment of treatment for drug addiction. Medical Decision Making.

[113] Henderson C, Knapp M, Fernandez JL, Beecham J, Hirani SP, Cartwright M (2013). Cost effectiveness of telehealth for patients with long term conditions (Whole Systems Demonstrator telehealth questionnaire study): Nested economic evaluation in a pragmatic, cluster randomised controlled trial. BMJ.

[114] Simon, J. K., Korrelboom, N., Kingdon, K., Wykes, D., Phiri, T., van der Gaag, P., Baksh, M., Steel, M. F. (unpublished manuscript). Cost-effectiveness of positive memory training (PoMeT) for the treatment of depression in schizophrenia: A within-trial economic evaluation. 10.3390/ijerph191911985 PMC9565889 36231292

[115] Botes R, Vermeulen KM, Ranchor AV, Buskens E (2018). Functional health state description and valuation by people aged 65 and over: A pilot study. BMC Geriatrics.

[116] Khan MA, Richardson J (2018). Variation in the apparent importance of health-related problems with the instrument used to measure patient welfare. Quality of Life Research.

[117] Simon J, Łaszewska A, Leutner E, Spiel G, Churchman D, Mayer S (2018). Cultural and linguistic transferability of the multi-dimensional OxCAP-MH capability instrument for outcome measurement in mental health: The German language version. BMC Psychiatry.

[118] Forder, J., Jones, K., Glendinning, C., Caiels, J., Welch, E., Baxter, K., et al. (2012). Evaluation of the personal health budget pilot programme.

[119] Glendinning, C., Challis, D., Fernández, J. L., Jacobs, S., Jones, K., Knapp, M., et al. (2008). Evaluation of the Individual Budgets Pilot Programme.

